# Structural and functional consequences of the STAT5B^N642H^ driver mutation

**DOI:** 10.1038/s41467-019-10422-7

**Published:** 2019-06-07

**Authors:** Elvin D. de Araujo, Fettah Erdogan, Heidi A. Neubauer, Deniz Meneksedag-Erol, Pimyupa Manaswiyoungkul, Mohammad S. Eram, Hyuk-Soo Seo, Abdul K. Qadree, Johan Israelian, Anna Orlova, Tobias Suske, Ha T. T. Pham, Auke Boersma, Simone Tangermann, Lukas Kenner, Thomas Rülicke, Aiping Dong, Manimekalai Ravichandran, Peter J. Brown, Gerald F. Audette, Sarah Rauscher, Sirano Dhe-Paganon, Richard Moriggl, Patrick T. Gunning

**Affiliations:** 1 Department of Chemical and Physical Sciences, University of Toronto Mississauga, 3359 Mississauga Road North, Mississauga, ON L5L 1C6 Canada; 2 Department of Chemistry, University of Toronto, 80 St. George Street, Toronto, ON M5S 3H6 Canada; 3 Institute of Animal Breeding and Genetics, University of Veterinary Medicine Vienna, 1210 Vienna, Austria; 4 Ludwig Boltzmann Institute for Cancer Research, 1090 Vienna, Austria; 5 Department of Physics, University of Toronto, 60 St. George Street, Toronto, ON M5S 1A7 Canada; 6 Dalriada Drug Discovery, University of Toronto Mississauga, 3359 Mississauga Road North, Mississauga, ON L5L 1C6 Canada; 7 Department of Cancer Biology, Dana-Farber Cancer Institute, Boston, MA 02215 USA; 8 Department of Biological Chemistry & Molecular Pharmacology, Harvard Medical School, 450 Brookline Avenue, Boston, MA 02215 USA; 9 Institute of Laboratory Animal Science, University of Veterinary Medicine Vienna, 1210 Vienna, Austria; 10 Unit of Laboratory Animal Pathology, University of Veterinary Medicine Vienna, 1210 Vienna, Austria; 11 Clinical Institute of Pathology, Department for Experimental and Laboratory Animal Pathology, Medical University of Vienna, 1090 Vienna, Austria; 12 Structural Genomics Consortium, University of Toronto, 101 College St., Toronto, ON M5G 1L7 Canada; 13 Department of Chemistry, York University, 327C Life Sciences Building, 4700 Keele Street, Toronto, ON M3J 1P3 Canada; 14 Medical University of Vienna, 1090 Vienna, Austria

**Keywords:** Biochemistry, Cancer

## Abstract

Hyper-activated STAT5B variants are high value oncology targets for pharmacologic intervention. STAT5B^N642H^, a frequently-occurring oncogenic driver mutation, promotes aggressive T-cell leukemia/lymphoma in patient carriers, although the molecular origins remain unclear. Herein, we emphasize the aggressive nature of STAT5B^N642H^ in driving T-cell neoplasia upon hematopoietic expression in transgenic mice, revealing evidence of multiple T-cell subset organ infiltration. Notably, we demonstrate STAT5B^N642H^-driven transformation of γδ T-cells in in vivo syngeneic transplant models, comparable to STAT5B^N642H^ patient γδ T-cell entities. Importantly, we present human STAT5B and STAT5B^N642H^ crystal structures, which propose alternative mutation-mediated SH2 domain conformations. Our biophysical data suggests STAT5B^N642H^ can adopt a hyper-activated and hyper-inactivated state with resistance to dephosphorylation. MD simulations support sustained interchain cross-domain interactions in STAT5B^N642H^, conferring kinetic stability to the mutant anti-parallel dimer. This study provides a molecular explanation for the STAT5B^N642H^ activating potential, and insights into pre-clinical models for targeted intervention of hyper-activated STAT5B.

## Abstract (web summary)

Hyper-activated STAT5B and its disease-causing variants are of interest as cancer drug targets. Here the authors combine cell based studies, X-ray crystallography, biophysical experiments and MD simulations to structurally and functionally characterize the STAT5B^N642H^ mutant found in aggressive T-cell leukemia and lymphomas and find that it has an increased affinity for self-dimerization.

## Introduction

The signal transducer and activator of transcription (STAT) family play key regulatory roles in cellular proliferation, metabolism, differentiation, and survival, and are expressed at a significantly higher level than corresponding cytokine receptor chains and upstream kinases^1^. The paradigm of STAT cytosolic-nuclear shuttling is initiated by cytokine/growth factor association with specific extracellular receptors. Unphosphorylated STATs form an anti-parallel dimer/monomer equilibrium, and upon efficient recruitment to the cognate receptor–kinase complex, are phosphorylated on a specific tyrosine residue, which promotes parallel dimerization by conformational rearrangement^2^. The activated STAT dimer translocates to the nucleus, initiating transcription of target genes and altering chromatin involved in loop formation in enhancer-promoter landscapes. Tyrosine dephosphorylation deters STAT activation and facilitates cytosolic shuttling/recycling, whereas impeding the STAT-activating Janus kinases (JAK) and cytokine receptor chains are intrinsically slower processes involving ubiquitination and degradation through suppressors of cytokine signaling (SOCS) ubiquitin ligases. This pleiotropic cascade is tightly regulated by a variety of ligands and effector proteins to provide extensive control over key cellular processes^3^. Hyperactivation of upstream effectors, including all JAK kinase family members, most childhood or adult cancer kinase translocations such as breakpoint cluster region-Abelson (BCR/ABL), and growth receptor hyperactivations such as FMS-like tyrosine kinase receptor-3 internal tandem duplication (FLT3-ITD) or v-erb-b2 erythroblastic leukemia viral oncogene homolog 2 (ERBB2), results in malignant transformation^1,2,4^. Leukemogenic driver mutations that arise during tumorigenesis have frequently been identified in the mutational landscape of hyper-activated STAT proteins, most prominent in T-cell prolymphocytic leukemia (T-PLL) or other mature T-cell neoplasms^5^. Notably, STAT5B^N642H^ was identified and validated as a severe oncogenic driver mutation^5–8^. We have shown that transgenic mice harboring STAT5B^N642H^ maintain a lower threshold to cytokine activation and rapidly develop aggressive mature CD8^+^ T-cell neoplasia^5^. This is consistent with in vitro models, suggestive of STAT5B^N642H^ promoting prolonged STAT phosphorylation and dimerization^7^. The therapeutic implications of patients identified with STAT5B^N642H^ include increased drug resistance, risk of relapse and poor outcomes^9^. Although novel therapeutic avenues have been proposed, including multimodal treatments with FDA-approved JAK inhibitors (ruxolitinib, baracitinib, and tofacitinib) in combination with Aurora kinase-targeting small molecules^10^, there are currently no clinical candidates targeting STAT5B or driver mutations thereof. This is partly due to the limited structural information available on the STAT proteins, as well as challenges associated with targeting transcription factors, including dynamic, non-contiguous interacting interfaces with shallow binding sites.

Herein, we further explore the aggressive transforming capacity of STAT5B^N642H^ revealing widespread organ infiltration of proliferative mature T-cells of not only CD8^+^ but also CD4^+^ and γδ T-cell subsets in the STAT5B^N642H^ transgenic mouse model. Notably, we demonstrate STAT5B^N642H^-driven transformation of γδ T-cells in an in vivo syngeneic transplant model, which may have utility as an important pre-clinical model for aggressive human γδ T-cell disease. Moreover, we reveal the crystal structures of human STAT5B and STAT5B^N642H^. In association with biochemical and structural data, our findings suggest that the SH2 domain of STAT5B^N642H^ adopts two unique conformations in the solid state and has an enhanced affinity for self-dimerization coupled with a marked resistance to dephosphorylation. These studies provide a rationale for understanding the molecular basis of the aggressive STAT5B^N642H^ driver mutation, as well as important structural information and insight into pre-clinical models for targeted therapeutic intervention of hyper-activated STAT5B.

## Results

### STAT5B expression and mutations in hematological cancers

**Fig. 1:**
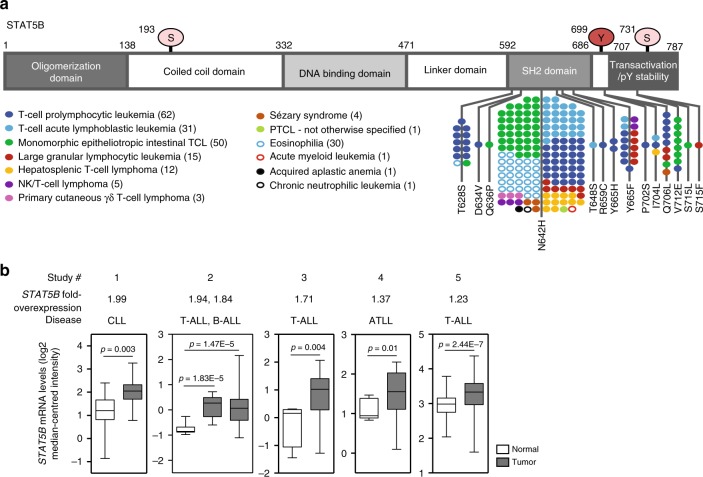
STAT5B is overexpressed and mutated at hot-spot residues in hematopoietic cancers. **a** Schematic depicting mutations found within the SH2 and C-terminal domains of human STAT5B in patients with various hematological malignancies. Each dot is representative of one patient. Numbers in brackets denote the number of patients reported to harbor any of the STAT5B mutations shown, for each disease entity. **b** Box plots showing human hematopoietic cancers with significant upregulation of *STAT5B* mRNA in tumor cells, compared with tissue-matched normal control cells. Data were extracted from the Oncomine database, from the following studies: 1 Haslinger Leukemia (chronic lymphocytic leukemia, CLL); 2 Andersson Leukemia (T-cell acute lymphoblastic leukemia, T-ALL; B-cell ALL); 3 Zhang Leukemia (Childhood T-ALL); 4 Choi Leukemia (chronic adult T-cell leukemia/lymphoma, ATLL); 5 Haferlach Leukemia (T-ALL). Representation: boxes as interquartile range, horizontal line as the mean, whiskers as lower and upper limits

Clinical and physiological studies have highlighted the functional dichotomy between the STAT5A and STAT5B gene products, and the increasingly influential role of STAT5B in tumorigenesis and proliferation. An important role for STAT5B, but not STAT5A, has been demonstrated for the pathogenesis of various disease-drivers, such as BCR/ABL^11,12^ and NPM-ALK^13^, where STAT5A was described as a tumor suppressor^14^. Notably, sequencing patient samples for gain-of-function (GOF) variants of STAT5B is becoming more frequent and has revealed multiple recurrent mutation hot-spots within the SH2 and C-terminal domains^5^ (Fig. 1a). Most notably, the N642H mutation has been detected in over 150 patients with various hematological malignancies, predominantly of T-cell origin. The most common T-cell diseases, representing over 60% of patients harboring the N642H mutation, include T-cell acute lymphoblastic leukemia (T-ALL), T-PLL, and monomorphic epitheliotropic intestinal T-cell lymphoma (MEITL; previously classified as enteropathy-associated T-cell lymphoma type II, EATL-II) (Fig. 1a). Interestingly, in addition to GOF mutations, mining of publicly available gene expression datasets also revealed significantly increased levels of *STAT5B* mRNA in patients with hematopoietic cancers of chronic lymphocytic leukemia (CLL), T-ALL, B-cell acute lymphoblastic leukemia (B-ALL), and adult T-cell leukemia/lymphoma (ATLL) origin (Fig. 1b). Indeed, increased expression of STAT5B protein was previously reported in CLL patients and correlated with poorer overall survival^15^. Furthermore, transgenic mice overexpressing STAT5B in the lymphoid compartment develop T-cell lymphomas^16^. Therefore, these patient data highlight the oncogenic potential of STAT5B in lymphoid neoplasia. As such, it is of interest to understand and characterize the molecular mechanisms of oncogenesis driven by hyper-activated STAT5B to devise better treatment strategies for these largely incurable diseases.

### STAT5B^N642H^ is a strongly aggressive oncogene

**Fig. 2:**
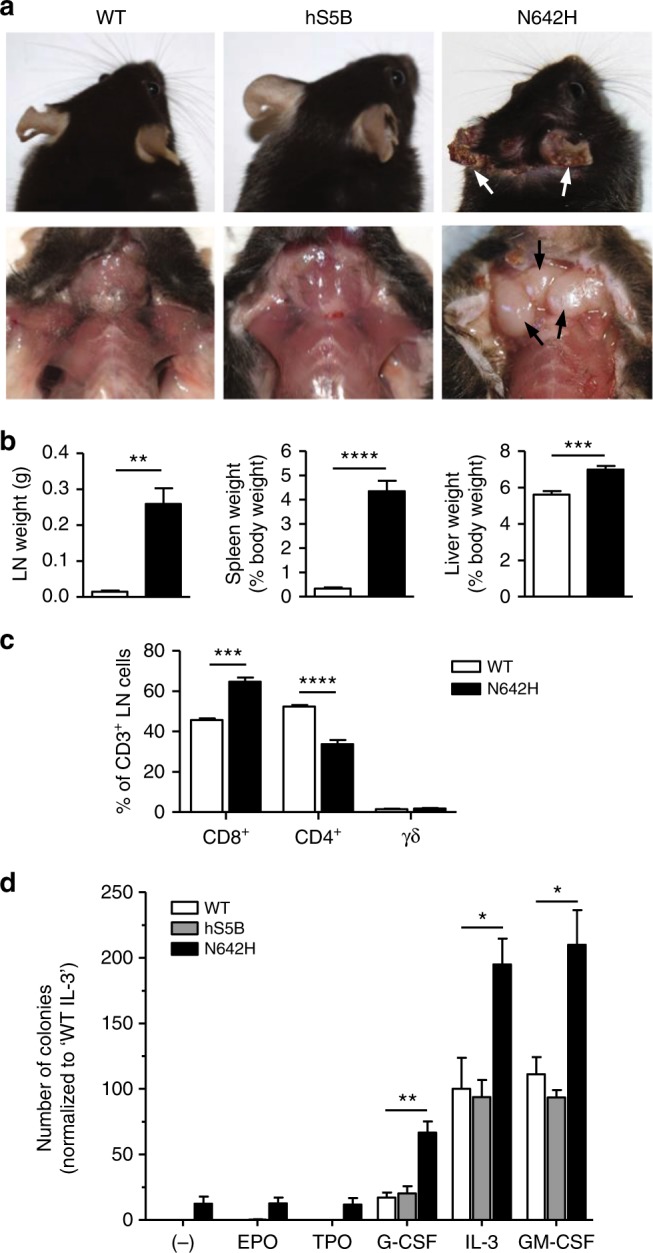
STAT5B^N642H^ promotes aggressive T-cell neoplasia and cytokine-independent cell growth. **a** Macroscopic images showing substantial skin lesions (upper panel; arrows) and lymphadenopathy (lower panels; arrows) observed in the STAT5B^N642H^ transgenic mice, compared with human STAT5B and wild-type (WT) control mice. **b** Axillary, brachial and inguinal LN, spleen, and liver weights were measured from 7- to 9-week-old WT (*n* = 4–6) and STAT5B^N642H^ (*n* = 5–8) transgenic mice. **c** Flow cytometric analysis of the percentage of CD8^+^, CD4^+^, or TCRγδ^+^ T-cells from the CD3^+^ cell population in the LNs of 7-week-old WT (*n* = 4) and STAT5B^N642H^ (*n* = 7) mice. **d** Colony-forming unit (CFU) assays using bone marrow cells from 7- to 10-week-old WT (*n* = 4), human STAT5B (*n* = 4) and human STAT5B^N642H^ (*n* = 5) mice, plated in duplicate in base methylcellulose in the presence (20 ng mL^–1^) or absence (-) of individual cytokines. Colonies were counted after 10 days. All data are graphed as mean ± SEM. *p < 0.05, **p < 0.01, ***p < 0.001, ****p < 0.0001, by unpaired two-tailed Student’s *t*-test (**b**, **c**) or one-way ANOVA with Bonferroni’s correction (**d**). Source data are provided as a Source Data file

Recently, we confirmed STAT5B^N642H^ as a driver mutation in T-cell neoplasia^5^. Transgenic mice expressing STAT5B^N642H^ within cells of the hematopoietic compartment rapidly succumb to mature T-cell lymphoma/leukemia, where the most dominant disease-causing cells are effector memory CD8^+^ cytotoxic T-cells^5^. We therefore wanted to further characterize these mice and assess their suitability as a pre-clinical model for human T-cell neoplasia. As previously observed, and in line with human patients suffering from mature peripheral and cutaneous T-cell neoplasias, STAT5B^N642H^ mice develop skin lesions resulting from disease-cell infiltration (Fig. 2a), as well as lymphadenopathy and splenomegaly (Fig. 2a, b). Interestingly, these mice also have significantly increased liver weight (Fig. 2b). Closer examination and immunophenotyping of different T-cell subtypes in the lymph nodes confirmed a shift in T-cell populations, with an increase in the proportion of CD8^+^ T-cells and a corresponding decrease in CD4^+^ T-cells (Fig. 2c). We also quantified γδ T-cells in the lymph nodes although no change was observed in the proportion of these relatively rare cells (Fig. 2c). Notably, the STAT5B^N642H^ mutation rendered bone marrow (BM) cells hypersensitive to various cytokines, resulting in significantly increased colony formation compared with BM cells from human STAT5B or wild-type mice (Fig. 2d). Additionally, in the absence of any cytokine, BM cells from STAT5B^N642H^ mice could still consistently form a small number of colonies, in contrast to BM cells from human STAT5B or  wild-type mice (Fig. 2d). These data demonstrate that the aggressive N642H mutation can support cytokine-independent proliferation and render cells hypersensitive to cytokine signaling.

### STAT5B^N642H^ transforms multiple T-cell subsets

**Fig. 3:**
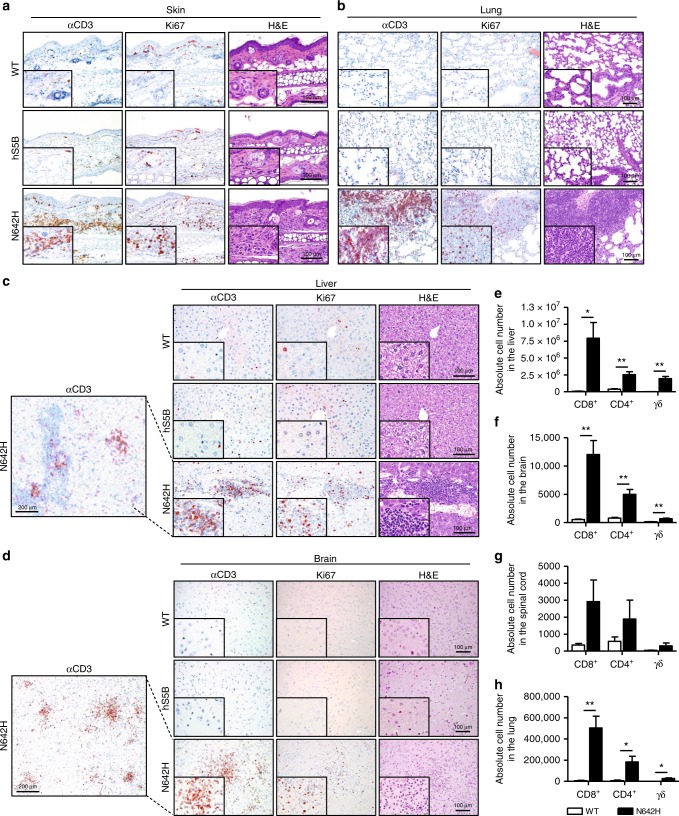
STAT5B^N642H^ transforms T-cell subsets, resulting in differential peripheral organ infiltration. **a**–**d** Histological analysis using CD3, Ki67, and H&E staining of the skin (**a**), lung (**b**), liver (**c**), and brain (**d**) of 7- to 9-week-old wild-type (WT), human STAT5B, and human STAT5B^N642H^ mice. Images are representative of three independent experiments. Original magnification: ×4 (left panels in **c** and **d**), ×20 and ×40 (insets), scale bars = 100 μm. **e**–**h** Flow cytometric analysis of the absolute cell numbers of CD8^+^, CD4^+^, or TCRγδ^+^ T-cells from the CD3^+^ cell population isolated from whole liver (**e**), brain (**f**), spinal cord (**g**), or lung (**h**) of 7-week old WT (*n* = 4) and STAT5B^N642H^ (*n* = 7) mice. All data are graphed as mean ± SEM. *p < 0.05, **p < 0.01, by unpaired two-tailed Student’s *t*-test. Source data are provided as a Source Data file

STAT5B^N642H^ has been detected in patients with diseases of varying immunophenotypes. In our STAT5B^N642H^ transgenic mouse model, mature CD8^+^ T-cells were identified as the predominant disease-initiating cells (Fig. 2c)^5^. We therefore sought to investigate the transforming and infiltrative capacity of other mature T-cell subsets, since patients with GOF mutations in the JAK1/3-STAT3/5B pathway also suffer from mature γδ or CD4^+^ T-cell diseases^5,17^. Upon immunohistological analysis of various peripheral organs from STAT5B^N642H^ mice, it was evident that these mice have severe, proliferative T-cell infiltrations into the skin and lung, as previously observed^5^, as well as in the liver and brain (Fig. 3a–d). These infiltrations are observed largely as foci spread throughout the organs. The infiltrating T-cells within different organs were subsequently immunophenotyped by flow cytometry. Notably, in addition to the dominant CD8^+^ T-cell infiltrations, CD4^+^ T-cells, as well as γδ T-cells were also found in increased numbers in various organs of STAT5B^N642H^ mice (Fig. 3e–h). Interestingly, where the proportion of infiltrating CD4^+^ T-cells was approximately half that of CD8^+^ T-cells across all organs examined, there was a notable increase in the proportion of infiltrating γδ T-cells into the liver of STAT5B^N642H^ mice compared with other organs (Fig. 3e–h).

These observations are interesting in the context of the different disease phenotypes of patients carrying the STAT5B^N642H^ mutation. It is clear that in our model, where STAT5B^N642H^ is expressed in all hematopoietic cells, CD8^+^ T-cells are most sensitive to transformation and therefore become highly proliferative and invasive. Surprisingly, we observed significant T-cell infiltration into the brain in the STAT5B^N642H^ mice, with the main T-cell phenotype being TCRβ^+^ CD8^+^ (Fig. 3d, f). T-ALL, one of the diseases with the highest number of STAT5B^N642H^-positive patients, is associated with infiltration into the central nervous system (CNS) upon relapse^18^. Overall, T-ALL patients with STAT5B^N642H^ were found to be at higher risk of relapse and have poorer event-free survival compared to other leukemia patients^6^. Furthermore, a patient from a primary CNS T-cell lymphoma cohort was reported to harbor the STAT5B^N642H^ mutation, and was diagnosed with a CD8^+^ CD4^−^ peripheral T-cell lymphoma, not otherwise specified (PTCL, NOS)^19^. An increasing number of large granular lymphocytic leukemia (T-LGLL) patients have also been reported to possess the STAT5B^N642H^ mutation (Fig. 1a), most of which had a CD4^+^ disease burden^20,21^, but one STAT5B^N642H^-positive CD8^+^ T-LGLL case was reported to be particularly aggressive and fatal^22^. T-PLL also has a mature predominantly CD4^+^ T-cell phenotype^23^, and in line with the considerable number of T-PLL patients carrying the STAT5B^N642H^ mutation (Fig. 1a), we also observed CD4^+^ T-cells infiltrating into peripheral organs in the STAT5B^N642H^ transgenic mice (Fig. 3e–h).

Hepatosplenic T-cell lymphoma (HSTCL) is predominantly a disease of γδ T-cell origin primarily affecting the liver, spleen and often BM^24^. The STAT5B^N642H^ mutation was detected in 33.3% (7/21) of patients from a HSTCL patient cohort^24^. It is therefore interesting that γδ T-cells from the STAT5B^N642H^ transgenic mice were observed in a relatively greater proportion in the liver compared with the other organs examined (Fig. 3e–h). It was previously reported that the diseased γδ T-cells in around one third of HSTCL patients harboring the N642H mutation were also CD8^+^ ^24^, and we indeed observed a large proportion of γδ-TCR^+^ CD8^+^ cells in the liver of the STAT5B^N642H^ mice (Supplementary Fig. 1). No STAT5B^N642H^ patients have been reported to carry γδ-TCR^+^ CD4^+^ cells, again consistent with our results (Supplementary Fig. 1).

### STAT5B^N642H^-transformed γδ T-cells reconstitute disease

**Fig. 4:**
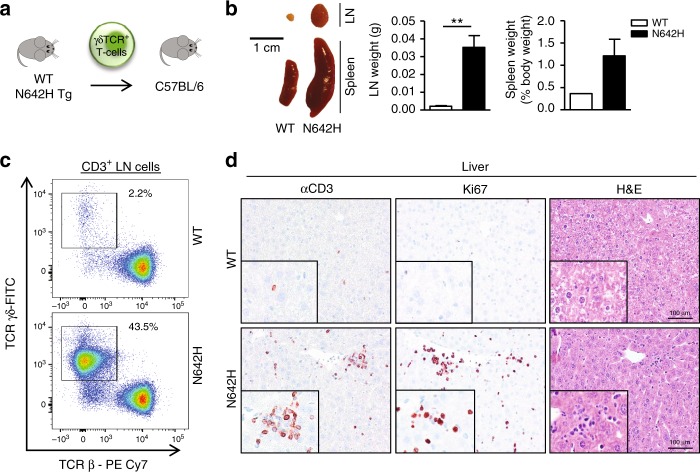
STAT5B^N642H^ transforms γδ T-cells, giving rise to aggressive γδ T-cell leukemia/lymphoma. **a** Schematic of isolation and transplantation of γδ T-cells from wild-type (WT) or hSTAT5B^N642H^ transgenic (Tg) mice into C57BL/6NRj recipient mice (WT *n* = 4, N642H *n* = 6) by tail vein injection. Two out of six N642H γδ T-cell transplant mice showed signs of disease burden at 3 months post transplantation, and they were sacrificed and analyzed along with one healthy WT γδ T-cell transplant mouse. **b** Macroscopic comparison of lymph node (LN) and spleen size from recipient mice transplanted with γδ T-cells from hSTAT5B^N642H^ (*n* = 2) or WT (*n* = 1) mice. Axillary, brachial, and inguinal LN and spleen weights were quantified and graphed as mean ± SEM, **p < 0.01 by unpaired two-tailed Student’s *t*-test (LN), or mean ± range (spleen). **c** Flow cytometric analysis of the percentage of γδ T-cells in the LNs of recipient mice transplanted with γδ T-cells from hSTAT5B^N642H^ (*n* = 2) or WT (*n* = 1) mice. **d** Histological analysis using CD3, Ki67, and H&E staining of the liver of recipient mice transplanted with γδ T-cells from hSTAT5B^N642H^ (*n* = 2) or WT (*n* = 1) mice. Original magnification: ×20 and ×40 (insets), scale bars = 100 μm. Source data are provided as a Source Data file

In order to verify whether the observed organ infiltration of these T-cell subsets represents true transformation by STAT5B^N642H^, we carried out a syngeneic transplant experiment as previously performed with neoplastic CD8^+^ T-cells from STAT5B^N642H^ mice^5^. Here, we chose γδ T-cells as our model transplant system because human T-cell diseases of γδ origin are among the most aggressive with extremely poor survival rates, and currently no pre-clinical models for these rare entities exist^17^. Strikingly, 3 months post-transplant of γδ T-cells from the STAT5B^N642H^ mice into immunocompetent recipients (Fig. 4a), an aggressive disease developed in a subset of the transplanted mice, characterized by lymphadenopathy and splenomegaly (Fig. 4b). Importantly, flow cytometric analysis of the lymph nodes of recipient mice confirmed a substantial increase in neoplastic γδ T-cells (Fig. 4c) but no considerable change in levels of CD8^+^ or CD4^+^ T-cells (Supplementary Fig. 2). Immunohistological analyses revealed infiltration of proliferative T-cells into the liver of recipient mice (Fig. 4d), consistent with observations in the STAT5B^N642H^ transgenic mice (Fig. 3c, e) and with the pathology observed in patients with aggressive γδ-driven HSTCL.

These data provide evidence of the transforming capacity and oncogenic nature of STAT5B^N642H^ in different T-cell subsets. Given that pre-clinical models for many of the rare and aggressive human T-cell neoplasia entities do not currently exist, as for the γδ T-cell subtypes, further development and characterization of such transplant models that recapitulate pathologies observed in these diseases will therefore be extremely valuable for testing much needed novel treatment strategies.

### Crystal structure of human STAT5B

**Fig. 5:**
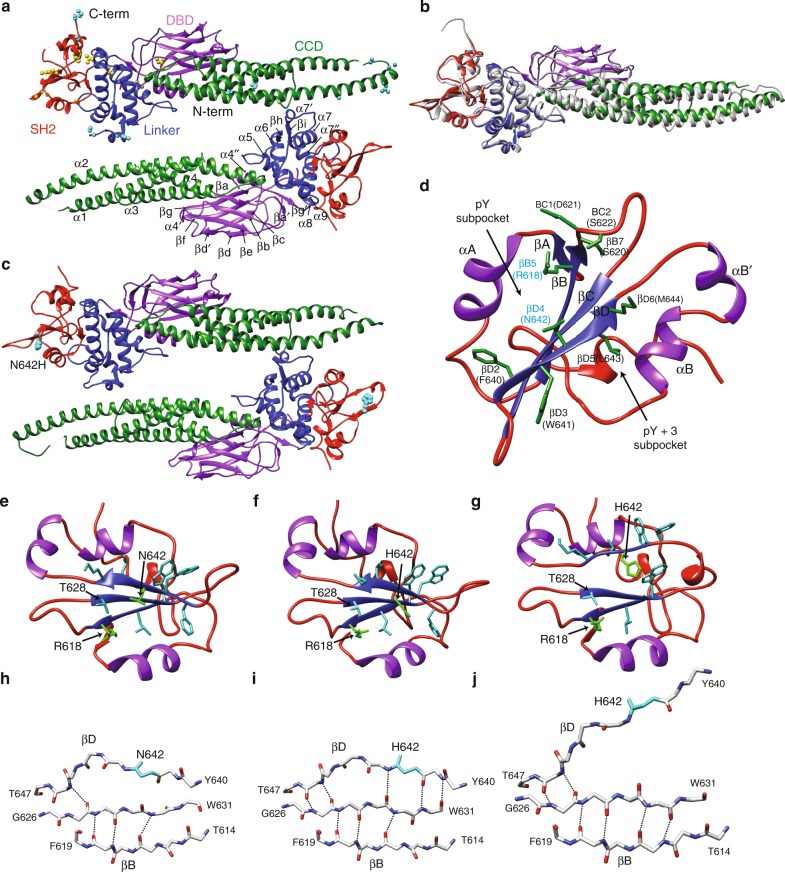
Structural architecture of human STAT5B and STAT5B^N642H^. **a** Crystal structure of the STAT5B unphosphorylated dimer illustrating the CCD (green), DBD (purple), linker domain (blue), and SH2 domain (red). The top monomer shows residue changes between human STAT5A and human STAT5B in yellow spheres, whereas differences between human STAT5A and murine STAT5A are shown in cyan spheres. Secondary structural elements are appropriately labeled on the bottom monomer. Nomenclature for structural elements in the CCD, DBD, and linker domain is established on precedents for STAT5A. **b** Overlay of human STAT5B with murine STAT5A (gray). **c** Crystal structure of the STAT5B^N642H^ unphosphorylated dimer with a similar color scheme as in **a**. The mutated N642H position is indicated by cyan spheres. **d** The SH2 domain of wild-type STAT5B with secondary structural elements labeled based on nomenclature for STAT-type SH2 domains. Different conformations of the STAT5B SH2 domain are shown for the wild-type (**e**) and mutant (**f**, **g**). The hydrogen bonding network for the β-sheet backbone in the SH2 domain for STAT5B (**h**) and STAT5B^N642H^ (**i**, **j**) are shown in two distinct conformations. Source data are provided as in the PDB: 6MBW (STAT5B) and 6MBZ (STAT5B^N642H^)

Given the aggressive nature of a single point mutation in transforming T-cell subsets, we sought to identify the molecular and structural basis of STAT5B^N642H^ activation. Currently, the structure of unphosphorylated mouse STAT5A has allowed for several computational studies of human STAT5B, modeled in the activated state based on crystal structures of activated STAT1/3 without N- and C-terminal domains^25,26^. These studies have provided insights into the distinct molecular mechanisms of action for each STAT5 gene product. In order to gain insight into the structural basis of the N642H mutation, as well as to validate modeling studies on the differences between STAT5A/B, we crystalized human STAT5B. We subjected full-length STAT5B (M1-S787) to a range of crystallization conditions, however, the protein crystals were not amenable to high-quality diffraction. Based on these results we prepared a more stable construct (A136-Q703, also referred to as the unphosphorylated STAT core fragment), that eliminated highly flexible regions of the protein and corresponded to similar domain boundaries employed for crystallization of STAT5A^27^, as well as other STAT proteins^28,29^. With these domain boundaries, human STAT5B was crystallized and diffracted to 3.29 Å resolution (Fig. 5a). The structure was solved via molecular replacement with mouse STAT5A (PDB: 1Y1U) and refined to R_work_ and R_free_ values of 0.266 and 0.306, respectively. Data collection and refinement statistics are summarized in Supplementary Table 1.

The overall architecture of human STAT5B is analogous to mouse STAT5A with a rmsd of 1.21 Å, and a rmsd of 1.89 Å and 2.03 Å, in comparison to mouse STAT3 and human STAT1 respectively. The canonical domains of the STAT family are all present with the coiled-coil domain (CCD, S138-T331), DNA-binding domain (DBD, F332-V470), linker domain (H471-D591), and SH2 domain (Q592-P685). Within these domain boundaries, there are 29 amino acid differences between human STAT5B and mouse STAT5A, which are predominantly located within disordered regions and the SH2 domain (Fig. 5a–c, Supplementary Fig. 3).

Archetypical STAT-type SH2 domains are comprised of an anti-parallel β-sheet created by three β-strands (conventionally labeled βB-βD) surrounded by three α-helices (αA, αB’, and αB)^30^, which are also observed in STAT5B (Fig. 5d). Conventional phospho-peptide binding occurs perpendicular to the central β-sheet with the phospho-Tyr accessing the N-terminal (pY) pocket on one face of the β-sheet and the flanking residues occupying the C-terminal (pY + 3) cavity on the opposite face^31^. The pY-binding pocket is positively charged and harbors a number of evolutionarily conserved residues, such as an invariant arginine (R618 on βB5), that form part of the complex phosphate coordination network. Notably, a critical His at the βD4 position that is found in 80 of 121 human SH2 domain-containing proteins^31^, is absent for all STAT proteins. The hydrophobic pY + 3 cavity serves as the peptide selectivity pocket and is predominantly formed by the βD strand and αB helix. As such, the surface-exposed βD strand controls access to both the phosphate-coordinating βB5 position and the selectivity residues located deeper in each of the N- and C- terminal pockets. Several of the variations between STAT5A and STAT5B occur within the βD strand and CD loop (Q636, M639, F640, M644), which ostensibly correlates to differences in peptide selectivity between the isoforms.

### N642 is a critical site for modulating STAT5B interactions

As illustrated in Fig. 1a, the SH2 domain is a hot-spot for several mutations with the most prevalent mutations occurring either on the βD strand (such as N642H) or in close proximity ( < 5 Å apart), suggesting a common mechanism of action. To investigate whether STAT5B^N642H^ perturbs the protein structure, we crystalized the mutant using the same domain boundaries as the  wild-type protein (A136-Q703). The crystal structure of STAT5B^N642H^ was solved to 3.21 Å with a similar space group to the wild-type protein (Supplementary Table 1 and Fig. 5c). The βD strand containing N642H was observed with excellent electron density and showed two distinct conformations (Fig. 5e–j). In one STAT5B^N642H^ conformation, the βD strand associates securely with the βC strand forming additional H-bonding partners (Fig. 5f, i), not observed in the wild-type conformation (Fig. 5e, h), thereby fully completing the anti-parallel β-sheet. In this conformation, the H642 likely coordinates the phosphate, similar to that observed in other SH2 domains such as Src and phosphatidylinositol-4,5-bisphosphate 3-kinase (PI3K). Interestingly, the substitution of N642H fulfills a similar role to the βD4 His which, as previously described, is a residue critical for peptide binding in these SH2 domain-containing proteins^31^, but absent in all STATs. Mutation of the βD4 His in these proteins abolishes peptide binding^31^, further suggesting STAT5B^N642H^ directly coordinates the phosphorylated species, consistent with previous computational predictions^26^. The second conformation showed the N642-containing βD strand dissociated from the βC and βB strands, resulting in an incomplete β-sheet (Fig. 5g, j). This partially completed β-sheet places the SH2 domain in an open, potentially inactivated, state. Alternatively, it is also possible that this conformation is more capable of activating STAT5B either through facilitating interactions with the C-terminal region and dimer interface or providing greater access to the pY pocket to increase phospho-ligand association kinetics. In any case, it is interesting that STAT5B^N642H^ can adopt either a hyper-activated or hyper-inactivated state.

**Fig. 6:**
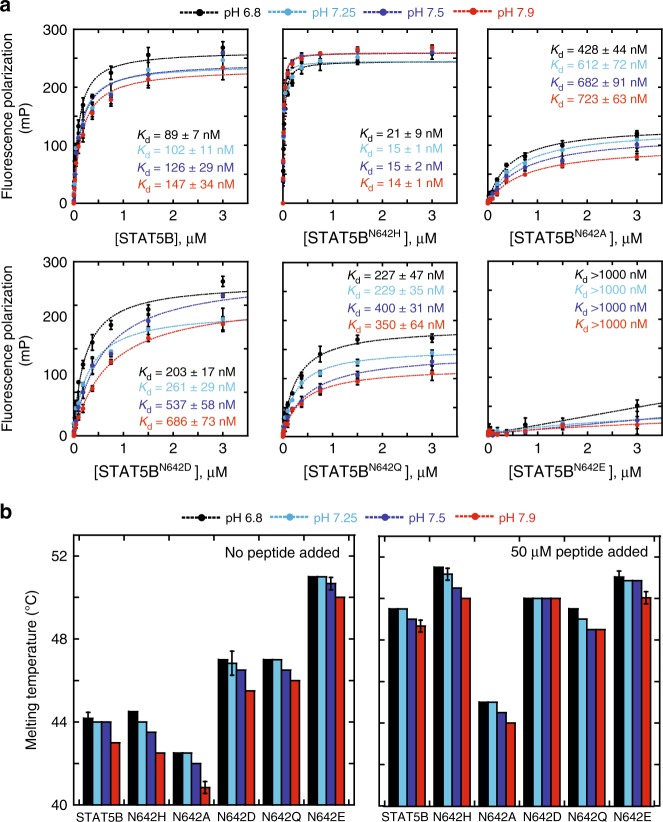
FP binding assays and denaturation temperatures of STAT5B /STAT5B^N642^ mutants. **a** The fluorescence polarization of 10 nM fluorescently labeled peptide was measured from samples containing increasing concentrations of wild-type/mutant STAT5B and the dissociation constants were determined from curve-fitting to a 1:1 protein-ligand model. The error bars show the mean ± SD from three independent titrations, and the *K*_d_ is reported with the error in the fit. **b** The denaturation temperatures of 2.5 µM STAT5B and STAT5B^N642^ mutants were determined at different pH values in the presence or absence of 50 µM peptide. The melting temperature is reported as the average from three independent samples and the error bars show the mean ± SD. Source data are provided as a Source Data file

To investigate whether conformational changes between STAT5B and STAT5B^N642H^ are the result of modified intramolecular or intermolecular interactions, we examined protein binding through fluorescence polarization (FP) with a fluorescein-labeled phospho-Tyr peptide derived from the EPO receptor^32^. Notably, the affinity of the phospho-peptide with the full-length STAT5B^N642H^ (*K*_d_ = 15 ± 1 nM) was ~6–7-fold higher than wild-type (*K*_d_ = 102 ± 11 nM, Fig. 6a). The change in peptide affinity is consistent with our structural findings of the SH2 domain pocket, as well as previous surface plasmon resonance (SPR) studies of STAT5B^N642H^ with phosphorylated peptide-dimer interface mimetics^7^. Interestingly, there is a significant increase in peptide binding with the corresponding STAT5B core fragments (*K*_d_ = 28.9 ± 1.9 nM for STAT5B, *K*_d_ = 2.6 ± 0.4 nM for STAT5B^N642H^, Supplementary Fig. 4). The tighter affinity of the core fragments (S136-Q703) compared to the full-length (M1-S787) is likely related to the increased accessibility of the SH2 domain in the absence of the C-terminal domain.

Previous molecular docking simulations have proposed favorable electrostatic interactions between the mutant imidazole and phosphate moiety of the peptide^7^. To examine the influence of electrostatics on this interaction, we varied the pH of the buffer and determined the dissociation constants (Fig. 6a). At lower pH values, stabilization of the interaction would be expected to increase the affinity for the peptide. While this is observed for  wild-type STAT5B, the binding affinity of STAT5B^N642H^ remains unchanged, suggesting the stabilizing interaction is not solely facilitated by electrostatics. To investigate the importance of sterics and electrostatics at this position, we generated a series of mutants and examined their phospho-peptide binding affinity. STAT5B^N642Q^ is analogous to the wild-type protein, with a slight perturbation arising from an extra methylene group in the side chain. However, the substitution of N642Q drastically reduced the binding affinity (*K*_d_ = 229 ± 35 nM). In contrast, removal of additional functional groups from the side chain with STAT5B^N642A^ also reduced the observed peptide binding (*K*_d_ = 612 ± 72 nM) further suggesting that this site does not tolerate changes in sterics. Contrastingly, STAT5B^N642D^ maintains the same shape of the wild-type asparagine but has opposite electrostatic properties. The negatively charged group was also found to have reduced peptide binding affinity (*K*_d_ = 261 ± 29 nM). Finally, STAT5B^N642E^, which possesses both steric bulk, as well as inferior electrostatic properties, completely abolished protein–peptide binding (*K*_d_ > 1 µM). All mutants also exhibited an increased peptide affinity at lower pH values, similar to wild-type STAT5B. STAT5B^N642D^ demonstrated the largest increase in peptide binding affinity at pH 6.8, compared to all the mutants generated, likely due to increased protonation and neutralization of the negative charge. In contrast to FP-binding experiments, thermodynamic denaturation profiles revealed the reverse trend in the stability of the STAT5B mutants (Fig. 6b). The electrostatic charge of STAT5B^N642D^ and the additional steric bulk of STAT5B^N642Q^ resulted in a significant stabilization of the protein (STAT5B *T*_m_ = 44 °C, STAT5B^N642D^ *T*_m_ = 47 °C, STAT5B^N642Q^ *T*_m_ = 47 °C). The combined effect of the changes in STAT5B^N642E^ greatly increased protein stability (STAT5B^N642E^ *T*_m_ = 51 °C). Consistent with the lack of binding observed in the FP experiments, the introduction of peptide did not increase the thermodynamic stability of STAT5B^N642E^ (*T*_m_ = 51 °C), but stabilized STAT5B^N642D^ (*T*_m_ = 50 °C) and STAT5B^N642Q^ (*T*_m_ = 49.5 °C). The collective implications of these results are interesting in the context of the two STAT5B^N642H^ conformations crystalized. Increased steric bulk and electronegative potentials at the N642 site promote a more stable and rigid STAT structure, likely the completed β-sheet domain conformation, but reduce the ability to bind phosphorylated peptides/species and possibly reduce overall STAT activity. The open conformation of the N642H-containing βD strand may allow for increased access to the peptide binding pocket. Once phospho-peptide binding occurs, the STAT5B^N642H^ may adopt a more stabilized structure further resulting in tighter binding. In the absence of phospho-peptide, STAT5B^N642H^ likely samples both conformations explaining the minimal changes in protein stability, but possibly favors the open state in the presence of peptide, allowing for tighter binding.

### STAT5B^N642H^ enhances anti-parallel dimer kinetic stability

**Fig. 7:**
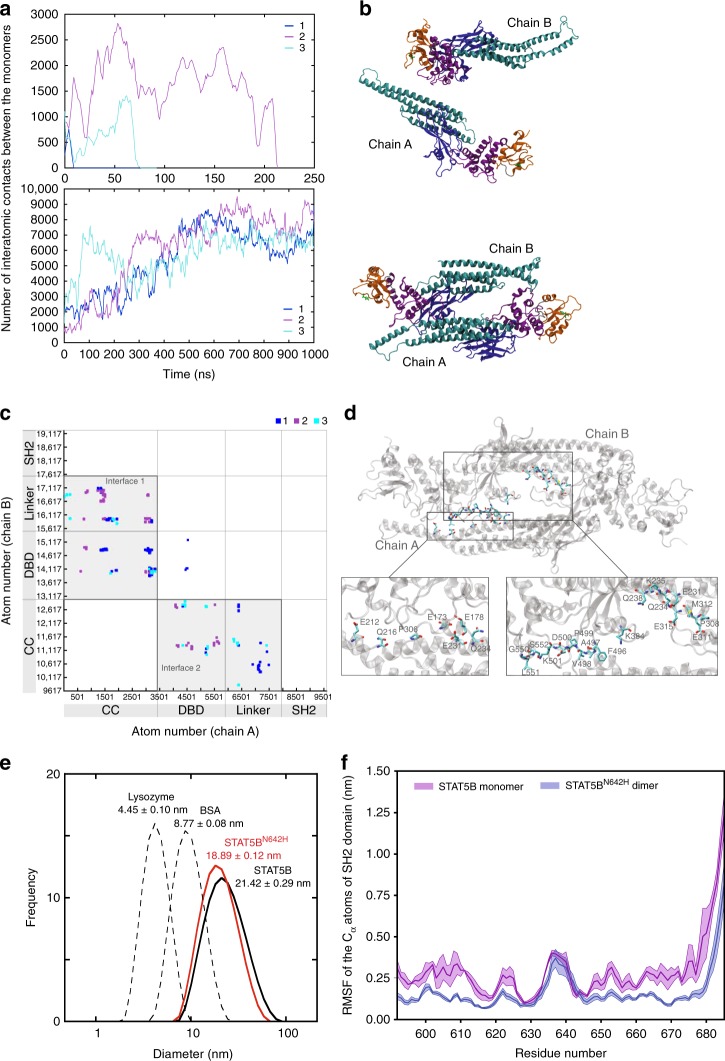
STAT5B and STAT5B^N642H^ dimer interfaces and flexibility of the SH2 domain. **a** The number of contacts formed between the monomers in the unphosphorylated anti-parallel dimeric structures of STAT5B (top) and STAT5B^N642H^ (bottom) as a function of simulation time. The three curves correspond to three independent simulations. Contacts are defined if any two atoms are within 6 Å; moving average over every 5 ns is plotted. **b** The structures of the STAT5B (system 3) at 75 ns depicting dimer dissociation (top) and STAT5B^N642H^ (system 1) at the end of 1 µs simulation (bottom). The color scheme in **b** corresponds to the individual domains: CCD, cyan; DBD, blue; linker domain, purple; SH2 domain, orange. Residue 642 is highlighted in green. **c** Inter-chain contacts formed over the 1 µs simulation, shown by the atom pairs within a minimum inter-atomic distance within a 6 Å cutoff. The boundaries of the individual domains are shown for clarity, and the two dimer interfaces are highlighted in gray boxes. **d** Sustained inter-chain contacts formed over the 1 µs simulation. The bottom panel demonstrates the hot-spot residues in licorice representation (chain A, left; chain B, right). See the simulation methods for the definition of the inter-chain contacts. Dynamic light scattering experiments in **e** show that STAT5B^N642H^ (50 µM) adopts a more closely packed dimer compared to STAT5B (50 µM). The values for diameter are reported ± SD as the average of three independent runs. **f** Root mean square fluctuation (RMSF) of the α-carbon atoms of the SH2 domain in STAT5B monomer and STAT5B^N642H^ dimer. The shading indicates statistical uncertainty. Source data are provided as a Source Data file

To examine the effects of the N642H mutation on the conformational mobility of STAT5B, we carried out molecular dynamics (MD) simulations of the unphosphorylated STAT5B and STAT5B^N642H^ anti-parallel dimers. Three independent simulations were performed for each dimer (Supplementary Movie 1–2). In all the tested systems, the STAT5B dimer exhibited marked instability and dissociated rapidly. In contrast, the STAT5B^N642H^ dimer remained intact over the entirety of the simulations (Fig. 7a, b). The STAT5B^N642H^ dimer was observed to be highly flexible, populating different dimer interfaces. The interactions between the CCD of chain A and DBD and linker domains of chain B (interface 1, Fig. 7c) form the most stable dimer interface, possibly responsible for the integrity of the anti-parallel dimeric structure. The second dimer interface, comprised of the CCD of chain B and the DBD and linker domains of chain A, is highly flexible, exchanging between multiple different conformations in each of the three simulation trajectories (interface 2, Fig. 7c). Further examination of the frequency of inter-chain contacts revealed several key residues in the CCD and linker domains responsible for the sustained inter-chain interactions in the anti-parallel STAT5B^N642H^ dimer (Fig. 7d and Supplementary Table 2). Since the CCD and linker domains contain the majority of these hot-spot residues, it appears that they play a more significant role in maintaining the stability of the interface than the DBD. Notably, these contacts are not close to the N642H mutation, suggesting a possible allosteric communication pathway between the CCD, linker and SH2 domains. These studies also suggest that STAT5B and STAT5B^N642H^ are regulated allosterically but differently and are influenced by the character of motions (collective and local) of the 642 residue, its environment and the interdependence of these motions.

To validate the in silico observations concerning anti-parallel dimer stability, we examined the hydrodynamic radius of STAT5B and STAT5B^N642H^ by dynamic light scattering (DLS) (Fig. 7e). Measurements of the particle size distribution of STAT5B via DLS suggest the full-length protein exists predominantly as a dimer (*d* = 21.42 ± 0.29 nm), which is also supported by gel filtration experiments (Supplementary Fig. 5). Similar experiments with STAT5B^N642H^ yielded a slightly smaller average particle size (*d* = 18.89 ± 0.12 nm), suggesting a more compact or stable anti-parallel dimer and reduced dissociation kinetics consistent with the MD simulations. (Fig. 7e).

Simulations of the anti-parallel STAT5B dimer were terminated upon observing dimer dissociation. To study the dynamics of the wild-type STAT5B protein, we carried out three independent simulations of the unphosphorylated STAT5B monomer. The root mean square fluctuation (RMSF) of the α-carbon atoms of the SH2 domain (Fig. 7f) indicates that N642H mutation leads to a more stable and rigid SH2 domain (except for residues 630 to 645, which are near the mutation site). The N642H mutation has little to no effect on the flexibility of CCD, DBD, and linker domains (Supplementary Fig. 6). We hypothesize that a more rigid SH2 domain may reduce the entropic cost of phospho-peptide binding and increase peptide-binding affinity.

### The STAT5^N642H^ parallel dimer resists dephosphorylation

**Fig. 8:**
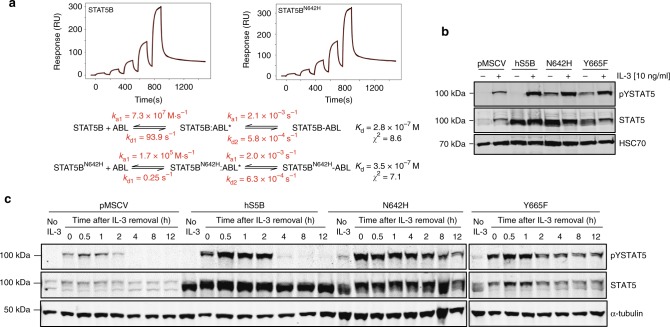
In vitro and in cellulo STAT5B/STAT5B^N642H^ phosphorylation and dephosphorylation. **a** SPR analysis of immobilized His-ABL1 kinase with increasing concentrations of either STAT5B or STAT5B^N642H^. The binding profile was fit to a two-state model with and the appropriate rate constants are shown. **b** Ba/F3 cells stably expressing human STAT5B, STAT5B^N642H^, STAT5B^Y665F^, or empty vector (pMSCV) were starved of IL-3 for 12 h and were then stimulated with 10 ng mL^−1^ IL-3 for 30 min. Activated, tyrosine phosphorylated STAT5 (pYSTAT5) and total STAT5 levels were analyzed by immunoblotting, and detection of HSC70 was performed as a loading control. **c** To assess phosphorylation kinetics, Ba/F3 stable cell lines described above were starved of IL-3 for 12 h (no IL-3) and were then stimulated with 10 ng mL^−1^ IL-3 for 30 min (0 h). Cells were subsequently washed to remove IL-3 and collected at various time points (0.5–12 h). pYSTAT5 and total STAT5 levels were analyzed by immunoblotting, and detection of α-tubulin was performed as a loading control. Blots are representative of two independent experiments. Source data are provided as a Source Data file

We probed the physiological relevance of tighter mutant-peptide interactions by examining the interaction of STAT5B and STAT5B^N642H^ with the upstream ABL1 kinase^33^ through SPR (Fig. 8a). ABL1 kinase was immobilized onto the chip surface and the response upon treatment with increasing concentrations of STAT5B or STAT5B^N642H^ was observed and corrected with blank injections. A significantly improved fitting (*χ*^2^_STAT5B _= 8.6, *χ*^2^_STAT5BN642H_ = 7.1) was obtained with a two-state kinetic model using the global data analysis option available within BiaEvaluation 3.0 software. This suggests that the ABL kinase-STAT interaction involves a two-step sequential process or conformational change. The kinetics of the first process appear to be substantially different between the wild-type and mutant protein, but the overall *K*_D_ values are not significantly different at a level that would be relevant at physiological concentrations. Additionally, no significant difference was observed between the total in vitro phosphorylation of the wild-type or mutant proteins upon detection via Pro-Q diamond gel staining (Supplementary Fig. 7).

To examine the molecular basis of dephosphorylation, kinetic experiments were performed to track the stability of the STAT5B activating phosphorylation site. To assess this, IL-3-dependent murine Ba/F3 cells were stably transduced with either human wild-type STAT5B, STAT5B^N642H^, STAT5B^Y665F^ (the second most frequent STAT5 mutation), or vector-GFP control. All stable cell lines were confirmed positive for GFP expression by flow cytometry (Supplementary Fig. 8A). Consistent with our observations, the N642H mutation promoted retention of Y699 phosphorylation in the absence of IL-3, which was also observed for STAT5B^Y665F^ but not for wild-type STAT5B overexpression or the endogenous protein (Fig. 8b). Examining phosphorylation kinetics, we found that STAT5B^N642H^ promoted the stability of Y699 phosphorylation up to 12 h upon removal of IL-3, whereas endogenous STAT5 and exogenous wild-type STAT5B were dephosphorylated by 4 h post IL-3 removal (Fig. 8c), a trend also consistent in murine 32D cells (Supplementary Fig. 8B) and previous reports in other cellular models^5,7^. These observations for both STAT5B^N642H^ and STAT5B^Y665F^ mutations may point to a convergent mechanism for STAT5 SH2 domain GOF mutants, which involve stabilization of the dimerization interface enabling prolonged activation.

It is interesting to note that the 32D cells displayed a considerably different immunoblot banding pattern for the GOF mutants, whereby they appeared to run predominantly as smaller molecular weight species (~80 kDa) but still seemed to influence the activation of full-length STAT5 proteins (Supplementary Fig. 8B). This phenomenon has been previously described for STAT5 proteins in 32D cells^34^, where it was proposed to not occur naturally but rather represent an artifact of cell extract processing due to the release of high levels of nuclear cathepsin G and proteolytic cleavage of STAT5, which is not observed in Ba/F3 cells. However, based on our observations, it is tempting to speculate that expression of oncogenic STAT5B mutants in these myeloid cells may specifically trigger activity of this protease as a negative regulatory mechanism to prevent cell transformation. This hypothesis could potentially explain the lower incidence of oncogenic STAT5B mutations found in myeloid diseases compared with T-cell neoplasia, but this will need to be further tested experimentally. Overall, in the context of in silico modeling, our in vitro and in cellulo experiments suggest STAT5B^N642H^ maintains a tighter dimer interface that resists dephosphorylation, allowing for prolonged stimulatory gene regulation as a consequence of a longer activation lifetime.

Overexpression of STAT5B is strongly correlated with the proliferation of multiple hematopoietic cancers and several oncogenic activating mutations have been identified. Here, we have revealed the structural origins and biochemical consequences of how an aggressive driver mutation such as STAT5B^N642H^ develops during cancer cell evolution to enhance phospho-Tyr:SH2 domain interactions and escape negative regulatory phosphatase attack. The STAT5B^N642H^ driver mutation was crystalized with the SH2 domain βD strand adopting two different conformations. These conformations may potentially result in hyperactivation or hyperinactivation of STAT5B, thereby suggesting a possible mechanism for the altered oncogenic activity. Moreover, we shed light on the transforming capacity of STAT5B^N642H^ in different T-cell lineages, particularly in γδ T-cells, allowing us to better understand and develop models for human disease. Overall, these studies offer insight into the molecular activation of STAT5B^N642H^, and the structural data, as well as pre-clinical in vivo models described here will assist with the future development of novel therapeutic strategies, which are urgently needed in rare but aggressive mature T-cell diseases with limited treatment options.

## Methods

### Human patient mutation and gene expression data

Data of patients harboring the STAT5B^N642H^ mutation were collected from previously published studies^6–8,19–22,24,35–52^. Gene expression analysis of *STAT5B* was performed for various human hematopoietic cancers using public gene expression datasets available from the Oncomine database^53^. For the analysis, the *p*-value threshold was set to 0.01, and both the fold-change and gene rank thresholds were set to all.

### Mammalian expression plasmids

Mammalian expression constructs containing FLAG-tagged human STAT5B or GOF variants in a pMSCV-IRES-GFP plasmid were previously generated^5,39^. The integrity and orientation of all complementary DNAs and the presence of point mutations was verified by Sanger sequencing.

### Transgenic animals and in vivo transplant experiments

Transgenic mice expressing either human STAT5B or STAT5B^N642H^ under the control of the *Vav1* promoter were previously described^5^. For transplant experiments, γδ T-cells from hSTAT5B^N642H^ mice or  wild-type littermates were isolated from pooled lymph node and spleen cell suspensions by FACS, and sorted cells were re-checked by flow cytometry for their purity. Cells (1.2 × 10^4^) were transplanted by lateral tail vein injection into nonirradiated C57BL/6NRj mice. Recipient mice were monitored daily and evaluated at the first sign of disease onset.

All animal studies were discussed and approved by the institutional ethics committee of the University of Veterinary Medicine Vienna, and animal experiment licenses were granted under GZ 68.205/0166-WF/V/3b/2015 and 68.205/0103-WF/V/3b/2015 (Austrian Federal Ministry of Science, Research and Economy). All animals were bred and maintained in a specific pathogen-free environment in the experimental mouse facility at the University of Veterinary Medicine (Vienna, Austria).

### Cell culture and generating stable cell lines

32D murine myeloid cells (#ACC 411) and Ba/F3 murine pro-B cells (#ACC 300) were purchased from the German Collection of Microorganisms and Cell Cultures (DSMZ). Cells were cultured in RPMI 1640 supplemented with 10% FBS, 2 mM l-glutamine, 10 U mL^–1^ penicillin-streptomycin (all from Gibco, Thermo Fisher Scientific) and 1 ng mL^–1^ murine Interleukin-3 (mIL-3; PeproTech). To generate retrovirus, Platinum-E retroviral packaging cells (Cell Biolabs) were transfected with plasmids using Lipofectamine 2000 (Invitrogen, Thermo Fisher Scientific), as per the manufacturer’s protocols. 32D and Ba/F3 cells were transduced with viral particles by spinfection, and pools of cells stably expressing the transgenes of interest were selected after 48 h by sorting for GFP-positive cells using fluorescence-activated cell sorting (FACS).

### Immunoblotting

Immunoblotting was performed using standard protocols. The following antibodies were used: polyclonal rabbit anti-phospho-STAT5 (Y694) (#71-6900; Invitrogen, Thermo Fisher Scientific; 1:1000), monoclonal mouse anti-STAT5 (#610191; BD Biosciences; 1:1000), monoclonal mouse anti-HSC70 (#sc-7298; Santa Cruz Biotechnology; 1:10000) and monoclonal mouse anti-α-tubulin (#sc-32293; Santa Cruz Biotechnology; 1:5000). Images of membranes were obtained using IRDye fluorescent secondary antibodies and an Odyssey CLx imaging system (LI-COR).

### Flow cytometry analyses

Whole body perfusion with phosphate-buffered saline (PBS) was performed on human STAT5B^N642H^ or wild-type mice, or transplant recipient mice, and organs were then collected and minced through a 70 μm cell strainer (BD Biosciences). For organ T-cell infiltration experiments, leukocytes were isolated using 40% and 78% Percoll gradients (GE Healthcare). Cells were then stained for FACS analysis using various antibodies purchased from eBioscience, Biolegend or BD Biosciences (see Supplementary Table 3 for antibody list). Counting beads (BioLegend) were added before acquisition to quantify absolute cell numbers. All analyses were performed on a FACSCanto II flow cytometer using FACSDiva software (BD Biosciences). Further analyses were performed using FlowJo software. The gating strategies employed are shown in Supplementary Figs. 9 and 10.

### Immunohistochemistry

Tissues were incubated for 24 h in 4% phosphate-buffered formaldehyde solution (Roti-Histofix; Carl Roth), dehydrated, paraffin-embedded, and cut (4-μm-thick sections). For immunohistochemical staining, heat-mediated antigen retrieval was performed in citrate buffer at pH 6.0 (Dako) and sections were stained with monoclonal rabbit anti-CD3 (#RM-9107; Thermo Fisher Scientific; 1:300) or monoclonal mouse anti-Ki67 (#NCL-Ki67p; Novocastra, Leica Biosystems; 1:1000) antibodies, using standard protocols. Images were obtained using an Olympus BX 53 LED light microscope with an Olympus SC50 camera.

### Hematopoietic colony assays

Bone marrow cells were isolated from the hind limbs of human STAT5B, human STAT5B^N642H^, or wild-type mice, and 5 × 10^4^ cells were plated in duplicate into 30 mm plates in base methylcellulose (#M3231; MethoCult; Stemcell Technologies) according to the manufacturer’s protocols, in the absence or presence of 20 ng mL^–1^ mIL-3, Erythropoietin (hEPO), Thrombopoietin (mTPO), granulocyte colony-stimulating factor (mG-CSF), or granulocyte-macrophage colony-stimulating factor (mGM-CSF). Colonies were incubated at 37 °C for 10 days and were counted manually using a light microscope.

### Protein expression

The gene (NCBI: NM_012448.3) encoding full-length human STAT5B (M1-S787) or the core fragment (A136-Q703) was synthesized and cloned into a pET-28b( + ) vector using restriction enzymes *Nhe*I and *Xho*I with a N-terminal His-SUMO tag. STAT5B^N642^ point mutations were generated through site-directed mutagenesis for both the full-length and core fragment. Molecular cloning was performed by GenScript. Protein expression and purification of all STAT5B proteins was carried out as described previously^32,54^. Briefly, BL21 (DE3) RILP cells were transformed with plasmid containing His-SUMO^-^STAT5B and single colonies were selected and cultured in 5 mL of lysogeny broth containing kanamycin (50 µg mL^–1^) and chloramphenicol (34 µg mL^–1^). The cultures were grown under continuous shaking at 37 °C for 3–4 h and used to inoculate 1 L of Super broth containing 10 mM MgSO_4,_ 0.1% (w/v) glucose, 3% (v/v) ethanol, kanamycin (50 µg mL^–1^) and chloramphenicol (34 µg mL^–1^). Following culture growth (OD_600_ = 2.0), the incubation temperature was reduced to 18 °C and the culture was induced with 0.5 mM IPTG. The cells were harvested after 18–20 h and stored at –80 °C. All reagents were purchased from BioShop.

### Protein purification and crystallization

STAT5B (wild-type and N642 mutants) protein purification was carried out by identical procedures. The cell pellets were lysed by sonication (Q55-QSonica) and the cell lysate was cleared by centrifugation. The fusion protein, His-SUMO-STAT5B, was isolated by Ni^2+^-nitrilotriacetic acid column chromatography (GE Healthcare). The fractions containing the STAT protein were treated with 0.1% (v/v) His-Ulp1 protease. His-Ulp1 protease was expressed and purified from *E. coli* using the same procedure as for STAT5B^55^. The plasmid containing Ulp1 protease, pFGET19_Ulp1, was a gift from Hideo Iwai (Addgene plasmid # 64697). The cleaved STAT protein was isolated by size exclusion chromatography (SEC650, Bio-Rad). The sample was dialyzed into 100 mM HEPES pH 7.4, 2% (v/v) glycerol. Protein concentrations were determined using a Pierce Bicinchoninic (BCA) Protein assay kit (Thermo Fisher Scientific).

For crystallography, the isolated STAT5B protein was dialyzed overnight into 20 mM HEPES pH 7.5, 200 mM NaCl, 1 mM TCEP and subsequently concentrated to 100–200 µM using 10 kDa MWCO concentrator (Amicon Millipore). Crystals of wild-type STAT5B protein were grown for 7–10 days in 200 mM lithium citrate, 20% (w/v) PEG3350. STAT5B^N642H^ crystals were grown in 200 mM lithium sulfate, 100 mM Tris-HCl pH 8.3, 25% (w/v) PEG3350, using 40–50 μM protein at 20 °C. Crystals were harvested from the drops using 0.05–0.1 mm Mounted CryoLoops–10 micron (Hampton Research) and stored in liquid nitrogen. Initial diffraction images for STAT5B and STAT5B^N642H^ were screened at Dana-Farber Cancer Institute and the Structural Genomics Consortium or York University, respectively.

### Data collection with structure solution and refinement

X-ray diffraction data for STAT5B were collected on NE-CAT beamline 24-ID-C at the Advanced Photon Source; data was collected on a Pilatus 6 M detector with 0.2 s exposure and 0.2° oscillation per frame (*λ* = 0.979 Å). Diffraction data for STAT5B^N642H^ was collected at the Canadian Macromolecular Crystallography Facility (CMCF), beamline 08ID-1, at the Canadian Light Source in Saskatoon, SK, Canada. Data for STAT5B^N642H^ were collected at a wavelength of 0.979 Å on a Pilatus 6 M detector with 0.3 s exposures and 0.3° oscillation per frame. Diffraction images were processed using the Xia2^56^ pipeline. The structure of STAT5B was solved by molecular replacement with Phaser^57^ using mouse STAT5A (PDB:1Y1U) as the search model; the STAT5^N642H^ structure used STAT5B as the search model. The structures were refined within Phenix^58–61^, with manual examination/rebuilding of |2*F*_o_| − |*F*_c_| and |*F*_o_| − |*F*_c_| maps using Coot^62^. Both structures exhibited two molecules per asymmetric unit and identical packing. Stereochemical quality of the final refined structures was done via MolProbity^63^, and deposited in the PDB as 6MBW (STAT5B) and 6MBZ (STAT5B^N642H^) with the corresponding statistics provided in Supplementary Table 1. Structures were visualized through the use of USCF Chimera^64^.

### Fluorescence polarization

Fluorescence polarization experiments were conducted using an Infinite M1000-Tecan Instrument with 384-well black plates (Greiner, medium binding). For fluorescence polarization experiments, the reaction samples (50 µL in volume) were prepared in 20 mM HEPES (pH 6.8, 7.25, 7.5, or 7.9), 50 mM NaCl, 0.1% (v/v) NP-40 substitute, 10% (v/v) DMSO with 10 nM fluorescently labeled (6-carboxyfluorescein or FAM) STAT5B-binding peptide (FAM-GpYLVLDKW, purchased from CanPeptide) and varying concentrations of STAT5B proteins. Each sample was incubated for 1 h at room temperature and the fluorescence polarization was detected at 530 nm following excitation at 470 nm (slit width of 5 nm in each case). The *K*_d_ was determined using the equation for binding as depicted below:

1 $${\mathrm{FP}} = {\mathrm{FP}}_{\mathrm{o}} + \frac{({\mathrm{FP}}_{\infty} - {\mathrm{FP}}_{\mathrm{o}})}{2\left[{\mathrm{Peptide}}_{\mathrm{Total}} \right]}\left[\vphantom{\sum^{x}}\left(\left[{\mathrm{Peptide}}_{\mathrm{Total}}\right] + \left[{\mathrm{STAT5}}_{\mathrm{Total}}\right] + K_{\mathrm{d}}\right)\right. \\ \hskip 10pt \left. - \, \sqrt{\left(\left[{\mathrm{Peptide}}_{\mathrm{Total}} \right] + \left[{\mathrm{STAT5}}_{\mathrm{Total}}\right] + K_{\mathrm{d}} \right)^{2} - \, 4\left(\left[{\mathrm{Peptide}}_{\mathrm{Total}} \right]\right)\left(\left[{\mathrm{STAT5}}_{\mathrm{Total}}\right]\right)} \right]$$

All experiments were repeated in triplicate to ensure reproducibility.

### Differential scanning fluorimetry (DSF)

DSF was performed using a BioRad CFX-96 Real Time PCR System, C1000 Thermal Cycler. The reactions (50 µL in volume) were performed with 100 mM HEPES buffer (pH 6.8, 7.25, 7.5, or 7.9) containing 150 mM NaCl, 0.2 mg mL^–1^ of wild-type or mutant STAT5B, and Sypro Orange (Invitrogen) at a final concentration of 5 × (1:1000 dilution). DSF was carried out by increasing the temperature by 0.5 °C/cycle from 20 to 80 °C, and fluorescence readings were collected at 30 s intervals. The fluorescence data and the second derivatives were collected and processed with the CFC Manager Software using the melt curve function of the software’s protocol editing tools. The temperature scan curves were fitted to a Boltzmann sigmoid function, and the *T*_m_ values were obtained from the midpoint of the transition. All experiments were repeated in triplicate to ensure reproducibility.

### Molecular dynamic simulations

The crystal structures of STAT5B and STAT5B^N642H^ dimers were subjected to all-atom MD simulations. The missing atoms in the crystal structures were added using Swiss-PDB Viewer^65^ and the missing loops were introduced using the loop model module of Modeler^66^. The resulting loop models were refined using the refine fast option of loop model. Three different structures for the loop regions were obtained as a result of loop modeling and refinement, which were used as initial structures in three independent simulations for each dimer. The simulation system in each case consisted of the dimer with charged termini (NH_3_^+^ at the N-terminus and COO^-^ at the C-terminus) in a rhombic dodecahedron box with 10 Å distance to all box edges, with CHARMM-modified TIP3P water molecules^67^ (~129,000 and ~134,750 water molecules for STAT5B and STAT5B^N642H^ dimers, respectively) and 150 mM NaCl. All simulations were carried out using GROMACS version 2016.5^68^. Energy minimization was carried out using the steepest descent algorithm. Prior to equilibration simulations, a restrained simulation was carried out for 2 ns with position restraints on the heavy atoms of the protein. Virtual sites were used^69^, allowing the use of an integration time step of 4 fs and periodic boundary conditions were applied. Short-range electrostatic interactions and van der Waals interactions were calculated with a cutoff of 0.95 nm. Long-range electrostatic interactions were evaluated using smooth Particle-mesh Ewald summation^70^ with 0.12 nm grid spacing and a fourth order interpolation. The Verlet cutoff scheme was used for neighbor searching. The bonds involving hydrogen atoms were constrained using the LINCS algorithm^71^ and water molecules were constrained using the SETTLE algorithm^72^. The velocity rescaling thermostat^73^ was used to maintain the temperature at 298 K. Equilibration simulations were carried out in the NPT ensemble for 10 ns using Berendsen pressure coupling^74^, followed by 10 ns simulation using the Parrinello-Rahman barostat^75^. Production runs were carried out in the NPT ensemble at 298 K and 1 bar using the Parrinello-Rahman barostat and the velocity rescaling thermostat. The CHARMM36m force field^76^ with the CHARMM-modified TIP3P water model was used for all of the simulations. Simulations were carried out for 1 µs for STAT5B^N642H^ dimers. In the simulations of the STAT5B dimers, the inter-monomer contacts were lost at 8 ns, 213 ns, and 75 ns, for systems 1, 2, and 3, respectively, and these simulations were terminated shortly after dimer dissociation was observed. To study the dynamics of the wild-type STAT5B protein, we carried out three independent simulations of the unphosphorylated STAT5B monomer by using the chain A from the corresponding STAT5B dimer systems as a starting structure. Simulations were carried out for 1 µs following the procedure described above. In all simulations, the simulation box was sufficiently large to avoid interactions between periodic images.

Visual molecular dynamics (VMD)^77^ was used for visualizing the simulation trajectories. The GROMACS utility mindist was used to calculate the inter-chain distances and contacts. Here, an inter-chain contact was defined as present if any two atoms are within a cutoff distance of 6 Å. The frequency of sustained inter-chain contacts was calculated using the VMD tool contactFreq.tcl. A sustained contact was defined when a contact between two non-hydrogen atoms within a cutoff of 4.5 Å is present for a total of 40% of the simulation time in at least one of the systems. The GROMACS utility rmsf was used to calculate the RMSF of the α-carbon atoms of the individual domains. For the unphosphorylated STAT5B monomer, the statistical uncertainty was calculated over three independent simulation systems. For the STAT5B^N642H^ dimer, RMSF was calculated for each SH2 domain individually, and the statistical uncertainty was reported over six SH2 domains in three independent systems. All-to-all RMSD of the non-hydrogen atoms of the SH2 domain was used as a convergence measure (Supplementary Fig. 11) and was calculated using the RMSD trajectory tool of VMD. Conformational states visited by the SH2 domain appear as blocks of regions with similar RMSD values along the diagonal. RMSD values in the off-diagonal regions are generally below 1 nm, indicating that conformational states are re-visited in independent simulations. This analysis shows that the conformational space of the SH2 domain is adequately sampled in both the unphosphorylated STAT5B monomer and STAT5B^N642H^ dimer systems. It should be noted that RMSD values are higher in STAT5B monomer systems, indicating higher flexibility in the wild-type monomer compared to the mutant dimer.

### Dynamic light scattering (DLS)

DLS experiments were performed on a Malvern Zetasizer NanoZS instrument with 50 µM samples of STAT5B and STAT5B^N642H^ (200 µL in volume) in 20 mM HEPES pH 7.4, 150 mM NaCl, 2% (v/v) glycerol. Standards of 1 mM Lysozyme (Bio-Shop) and Bovine Serum Albumin (BSA, Sigma-Aldrich) were also prepared in the same buffer. All samples were centrifuged for 10 min at 14,800 rpm and the data were collected in a quartz cuvette (1 cm path length). The hydrodynamic radius was determined from the average of the frequency distribution of particle sizes in three independent experiments for each sample.

### Surface plasmon resonance (SPR)

SPR experiments were performed at room temperature using the Biacore T200 (GE Healthcare) and a dextran-coated gold sensor chip functionalized with nitrilotriacetic acid (Series S Sensor Chip NTA, GE Healthcare). His-tagged ABL1 was immobilized using Ni^2+^-NTA affinity. Measurements were performed in PBS running buffer, including 0.05% Tween 20 and 50 µM EDTA. The surfaces of flow cells two (Fc2) and one (Fc1) were conditioned with 350 mM EDTA (GE Healthcare) for 60 s at a flow rate of 30 µL min^–1^. Fc2 was then prepared for ligand immobilization with 0.5 mM NiCl (GE Healthcare) injection at 30 µL min^–1^. Fifty nanomolar His-tagged ABL1 kinase was injected onto to FC2 at 30 µL min^–1^ until at least 400 RU was reached, and immobilized ABL1 kinase was allowed to stabilize for 30 min. Single cycle kinetics was used to collect kinetic data. Full-length STAT5B or STAT5B^N642H^ in running buffer was injected over the two flow cells at varying concentrations (0.0124, 0.0370, 0.111, 0.333, 1 µM) at a flow rate of 30 µL min^–1^ and a contact time of 120 s. The complex was allowed to dissociate for 600 s. The surfaces were regenerated with a 120 s injection of 350 mM EDTA, followed by a 120 s injection of 500 mM imidazole. Data were double referenced by subtracting binding responses from buffer solution flown over the active (Fc2) and reference (Fc1) flow cell. Data were collected at a rate of 10 Hz and fit to a two-state kinetic model using the global data analysis option available within BiaEvaluation 3.0 software.

### Statistics

Weight measurements and flow cytometry data are reported as mean ± SEM and differences were assessed for statistical significance by unpaired, two-tailed Student’s *t*-tests. Colony quantifications are reported as mean ± SEM and statistical differences were assessed by one-way ANOVA with Bonferroni’s correction. A *p*-value of <0.05 was considered statistically significant.

### Reporting summary

Further information on research design is available in the Nature Research Reporting Summary linked to this article.

## Supplementary information

Supplementary Information

Description of Additional Supplementary Files

Supplementary Movie 1

Supplementary Movie 2

Reporting Summary

Source Data

## Data Availability

The atomic coordinates and structure factors (STAT5B PDB code: 6MBW, STAT5B^N642H^ PDB code: 6MBZ) have been deposited in the Protein Data Bank, Research Collaboratory for Structural Bioinformatics, Rutgers University, New Brunswick, NJ (http://www.rcsb.org/). The source data underlying Figs. 2b–d, 3a-h, 4b-d, 6a-b, 7d, 8a-c, and Supplementary Figs. 1, 2, 4, 5, 6, 7, and 8a, b are provided as a Source Data file. Additional data for Figs. 2a–d, 4d are available online [10.5281/zenodo.2654892]. Other data are available from the corresponding authors upon reasonable request.
