# Supplementary material for: Structural and functional consequences of the STAT5BN642H driver mutation
Source: Nat Commun. 2019 Jun 7;10:2517. doi: 10.1038/s41467-019-10422-7 (PMC6555848; doi:10.1038/s41467-019-10422-7)
Supplement: Supplementary file 1 — Supplementary Information [file 41467_2019_10422_MOESM1_ESM.pdf]

Structural and Functional Consequences of the STAT5B^{N642H} Driver Mutation

E. D. de Araujo, F. Erdogan, H.A. Neubauer et al.

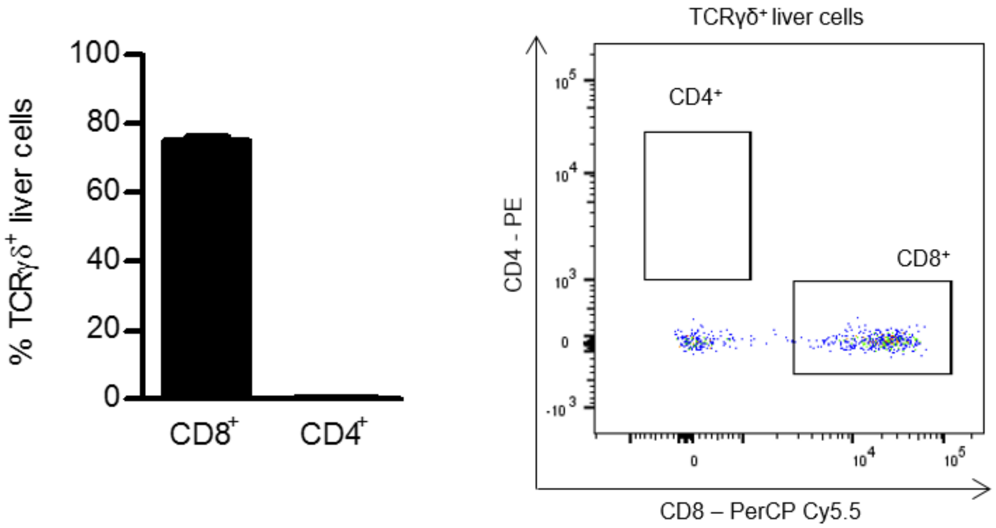

Supplementary Figure 1:

Flow cytometric analysis of the percentage of $CD8^+$ and $CD4^+$ T-cells from the $CD3^+$ $TCR\gamma\delta^+$ T-cell population isolated from the liver of 7-week old $STAT5B^{N642H}$ mice (n = 7). Data are graphed as mean ± SEM, and a representative FACS plot is shown.

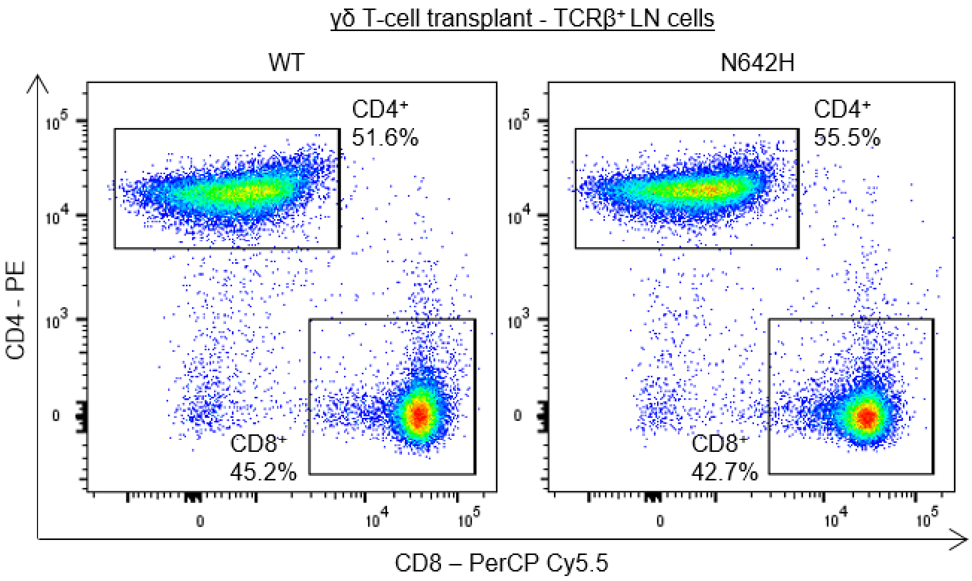

Supplementary Figure 2:

Flow cytometric analysis of the percentage of $CD8^+$ and $CD4^+$ T-cells within the $TCR\beta^+$ T-cell population from the lymph nodes (LN) of recipient mice transplanted with γδ T-cells from $STAT5B^{N642H}$ (*n* = 2) or wild type (WT) (*n* = 1) mice. Representative FACS plots and percentages of cell populations are shown.

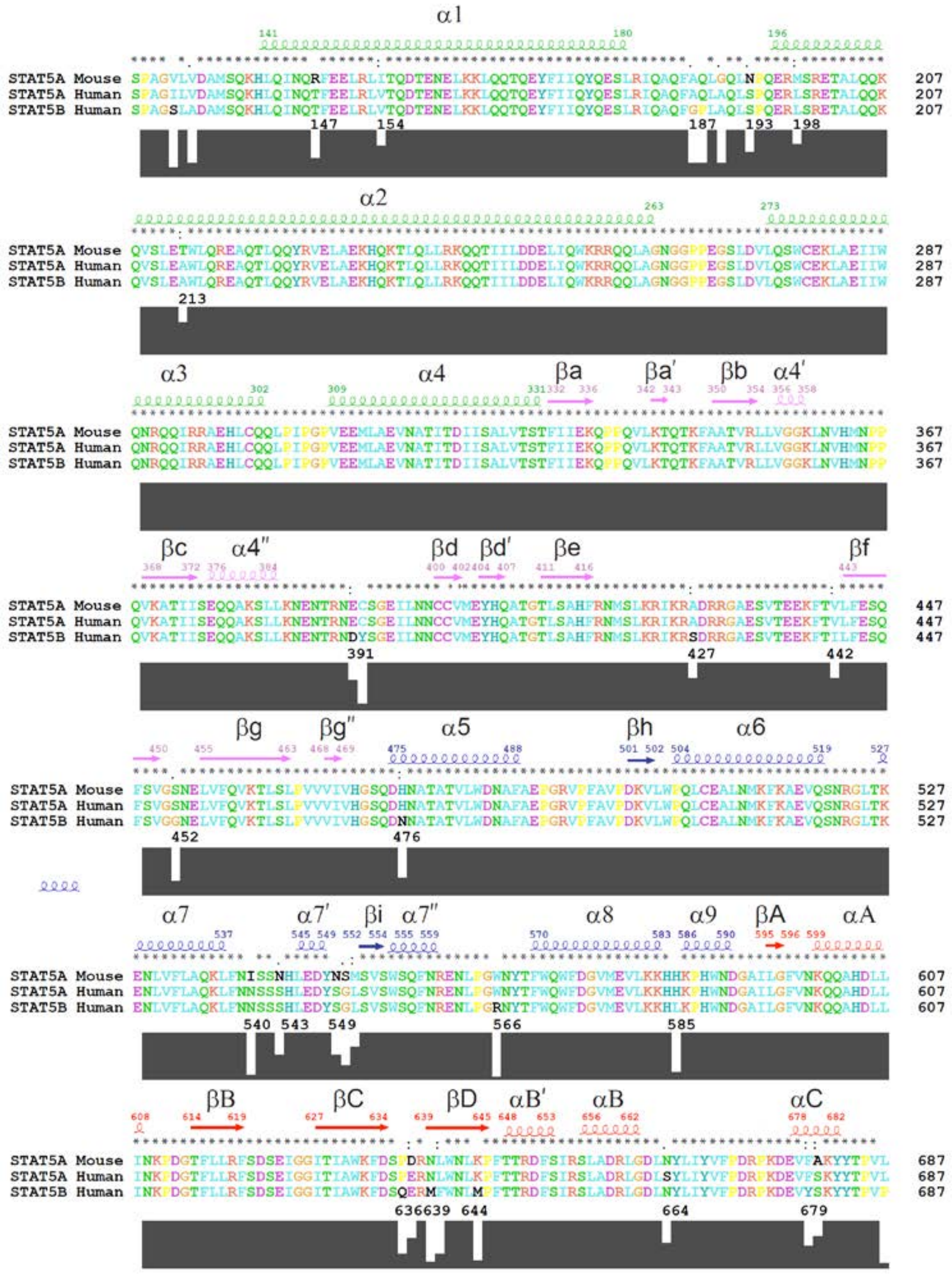

Supplementary Figure 3:

Sequence alignment of murine STAT5A, human STAT5A and human STAT5B generated using ClustalX2.1. The crystal structures for murine STAT5A and human STAT5B have been solved and the secondary structural elements identified for STAT5B are shown at the corresponding positions above the alignment and colored according to the appropriate domain (CCD green; DBD magenta; Linker domain blue; SH2 domain red). Secondary structural elements are labelled based on STAT5A for the CCD, DBD and Linker domains and the SH2 domain is labelled according to conventional nomenclature for SH2 domains.

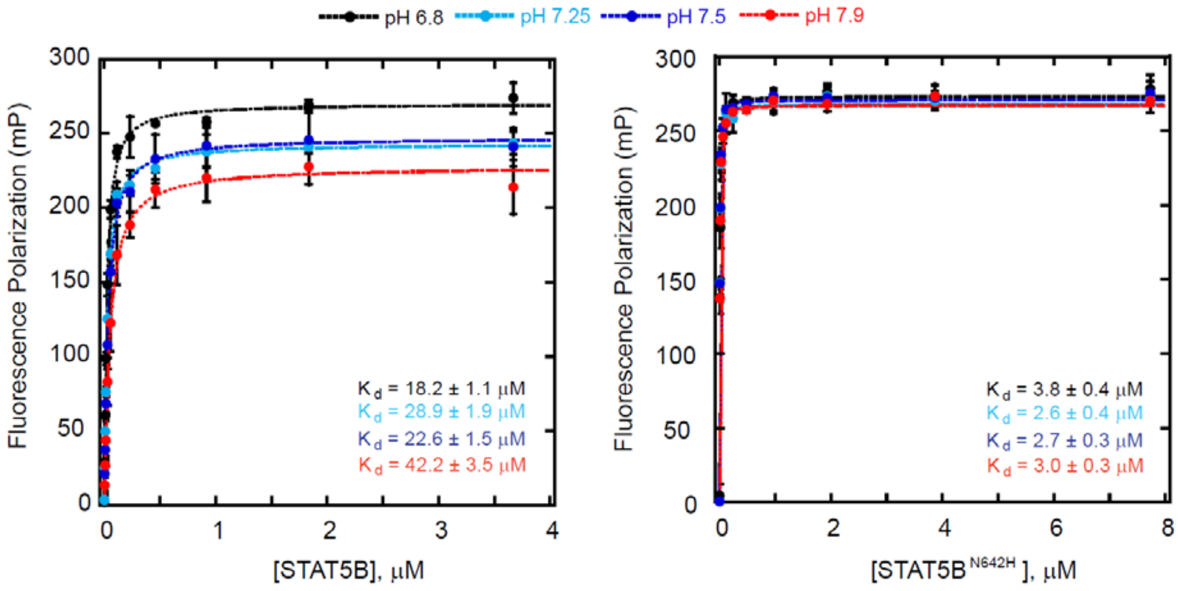

Supplementary Figure 4:

Fluorescence polarization binding assays of STAT5B and STAT5B^{N642H} (core fragments) over a range of pH values. The fluorescence polarization of 10 nM FAM-labelled peptide was measured from samples containing increasing concentrations of wild type/mutant STAT5B and the dissociation constants were determined from curve-fitting to a 1:1 protein-ligand model. The data is shown with ± SD from three independent measurements and the K_d values are shown with the error in the fit.

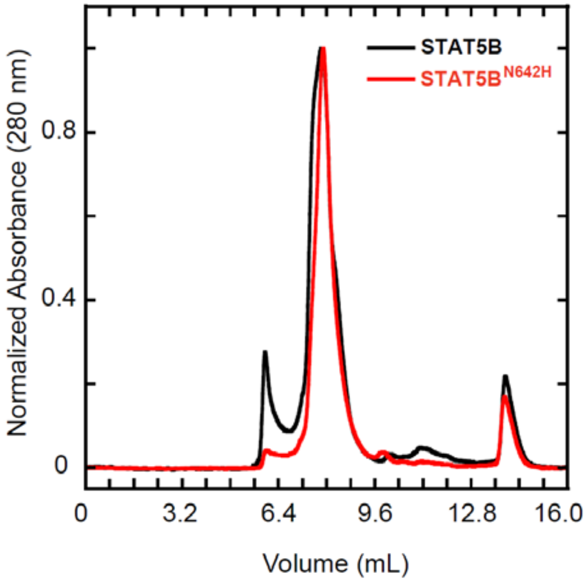

Supplementary Figure 5:

Gel filtration UV-traces of STAT5B and STAT5B^{N642H} (30 μM) in 20 mM HEPES pH 7.4, 150 mM NaCl, 2% glycerol, 2 mM DTT were obtained using the Wyatt WTC030S5 column on AKTA FPLC (GE Healthcare).

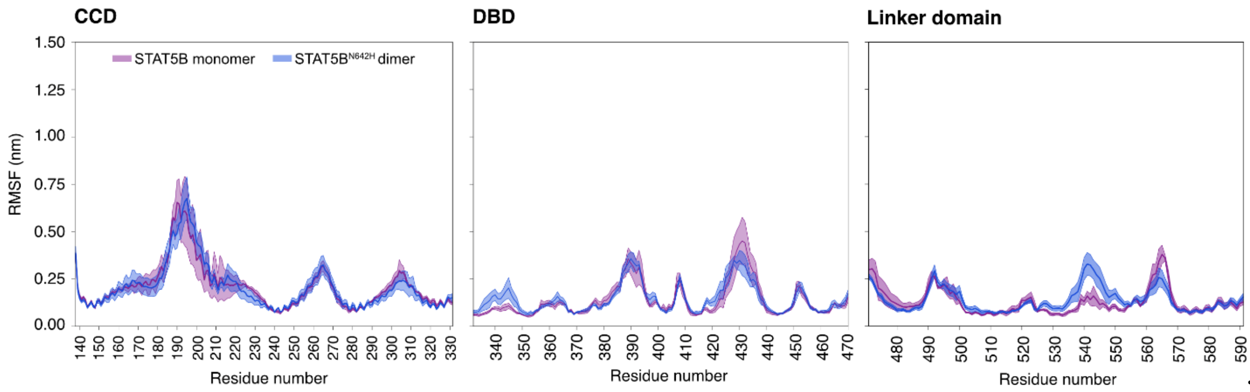

Supplementary Figure 6:

Comparison of the root mean square fluctuation (RMSF) of the α-carbon atoms of the CCD, DBD, and linker domain between the unphosphorylated STAT5B monomer and the antiparallel STAT5B^{N642H} dimer. The y-axis ticks and legend apply to all sub-figures. Shading indicates statistical uncertainty.

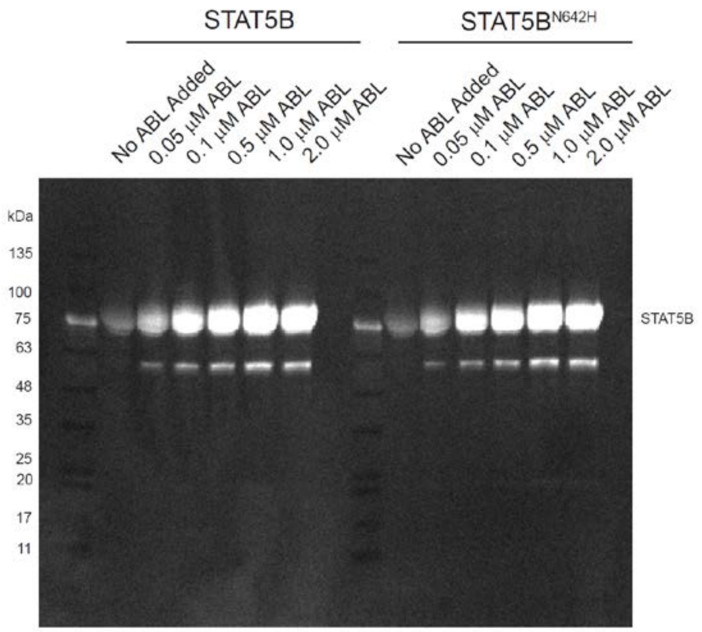

Supplementary Figure 7:

Phosphorylation of STAT5B and STAT5B^{N642H} was performed with 10 µM STAT5B (wild type or mutant) and varying concentrations of ABL kinase (0.05–2.0 µM) in the presence of 10 mM MgATP. Following 1 h incubation at 30 °C, the reaction was terminated by the addition of SDS loading buffer (50 mM Tris pH 6.8, 2 % [v/v] sodium dodecyl sulfate, 0.1 % (w/v) bromophenol blue, 100 mM DTT and 100 mM β-mercaptoethanol). All samples (7.5 µg) were loaded and resolved by SDS-PAGE and stained with Pro-Q diamond to detect the phosphorylated species. No significant differences were observed between the wild type and mutant with respect to the extent of phosphorylation observed.

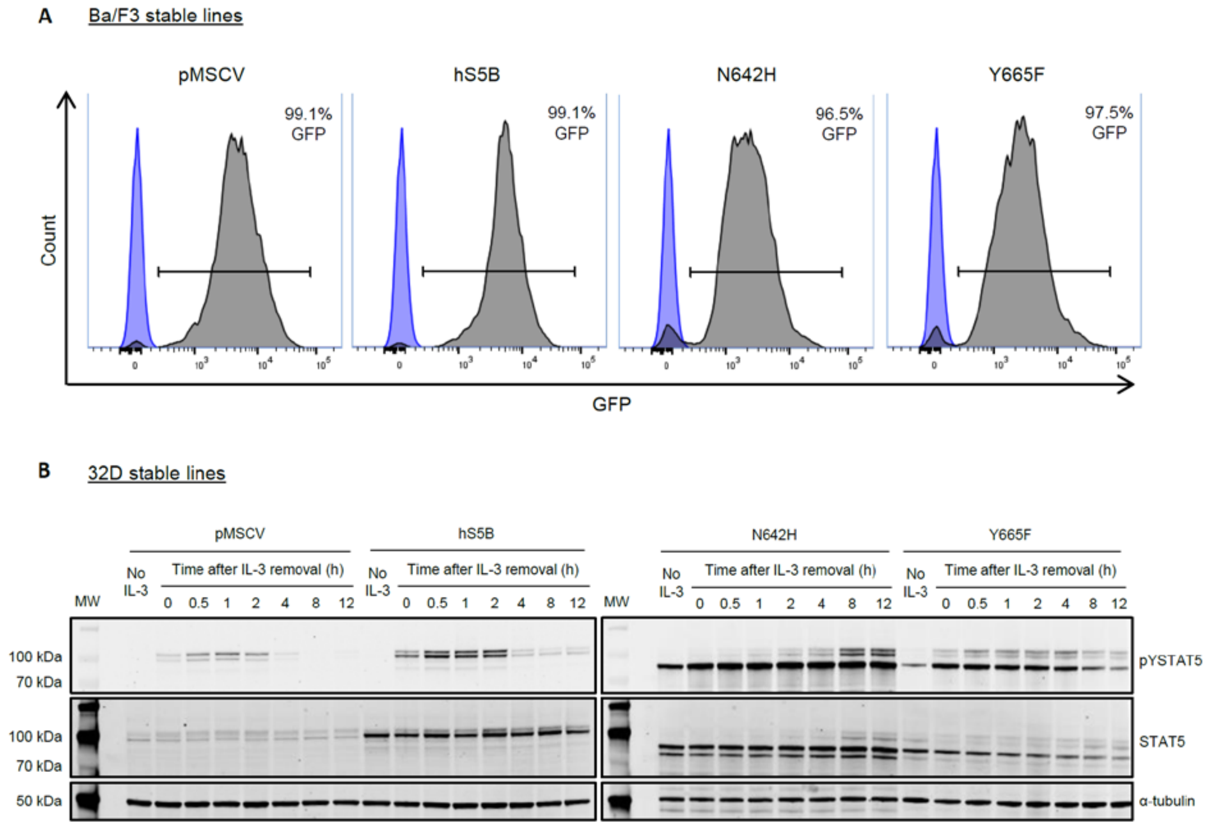

Supplementary Figure 8:

(**A**) Flow cytometric analysis of the percentage of GFP^{+} cells in the Ba/F3 stable cell line pools (grey) generated by retroviral transduction with empty vector (pMSCV-IRES GFP), human wild type STAT5B, STAT5B^{N642H} or STAT5B^{Y665F}. In each FACS plot, negative GFP-expression in parental Ba/F3 cells is depicted in blue. (**B**) 32D cells stably-expressing human STAT5B, STAT5B^{N642H}, STAT5B^{Y665F} or empty vector (pMSCV) were starved of IL-3 for 12 h (no IL-3) and were then stimulated with 10 ng·mL^{-1} IL-3 for 30 min (0 h). Cells were subsequently washed to remove IL-3 and collected at various time points (0.5 – 12 h). Activated, tyrosine phosphorylated STAT5 (pYSTAT5) and total STAT5 levels were analysed by immunoblotting, and detection of α-tubulin was performed as a loading control. Blots are representative of three independent experiments, using two independently generated sets of cell lines.

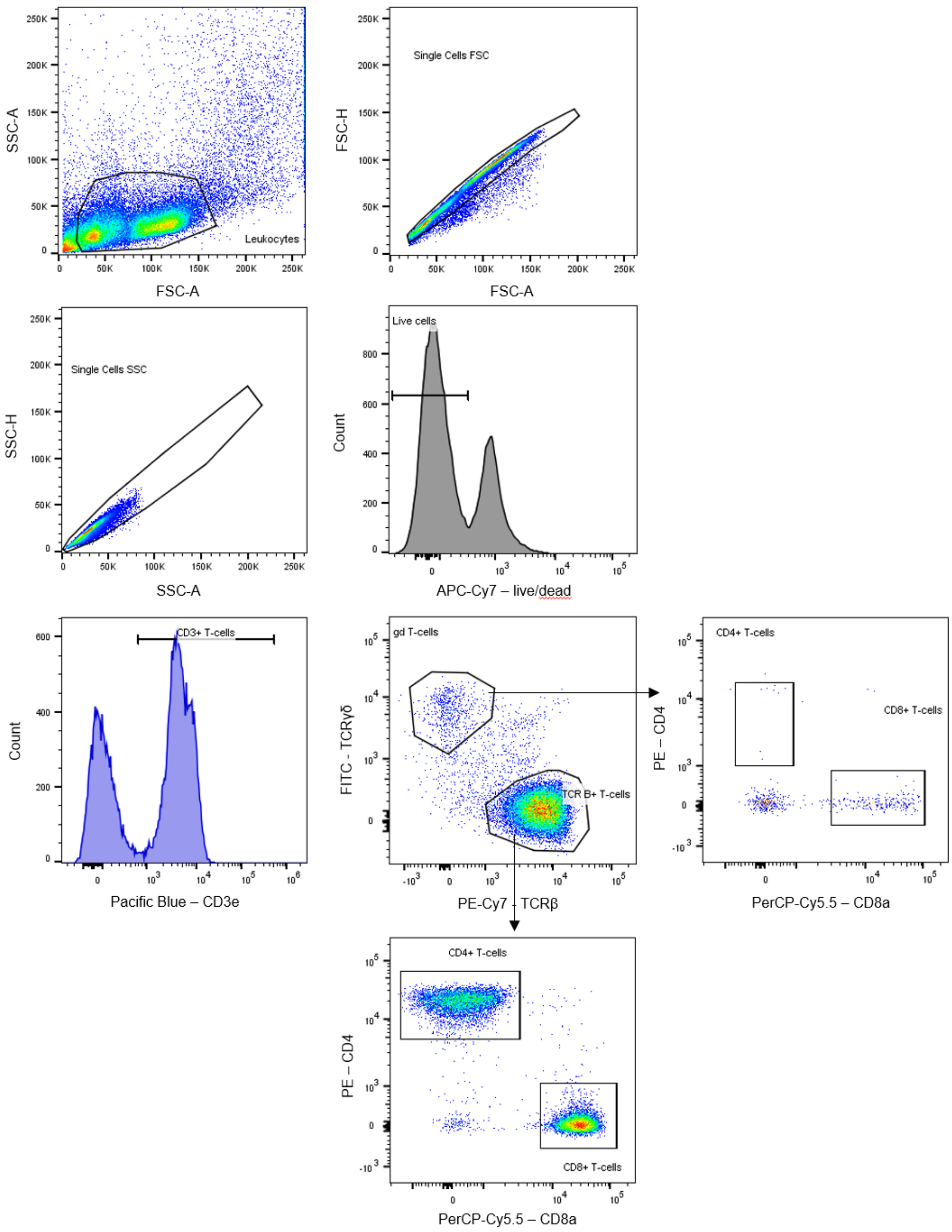

Supplementary Figure 9:

FACS gating strategy used for organ infiltration experiments in Fig. 3E-H.

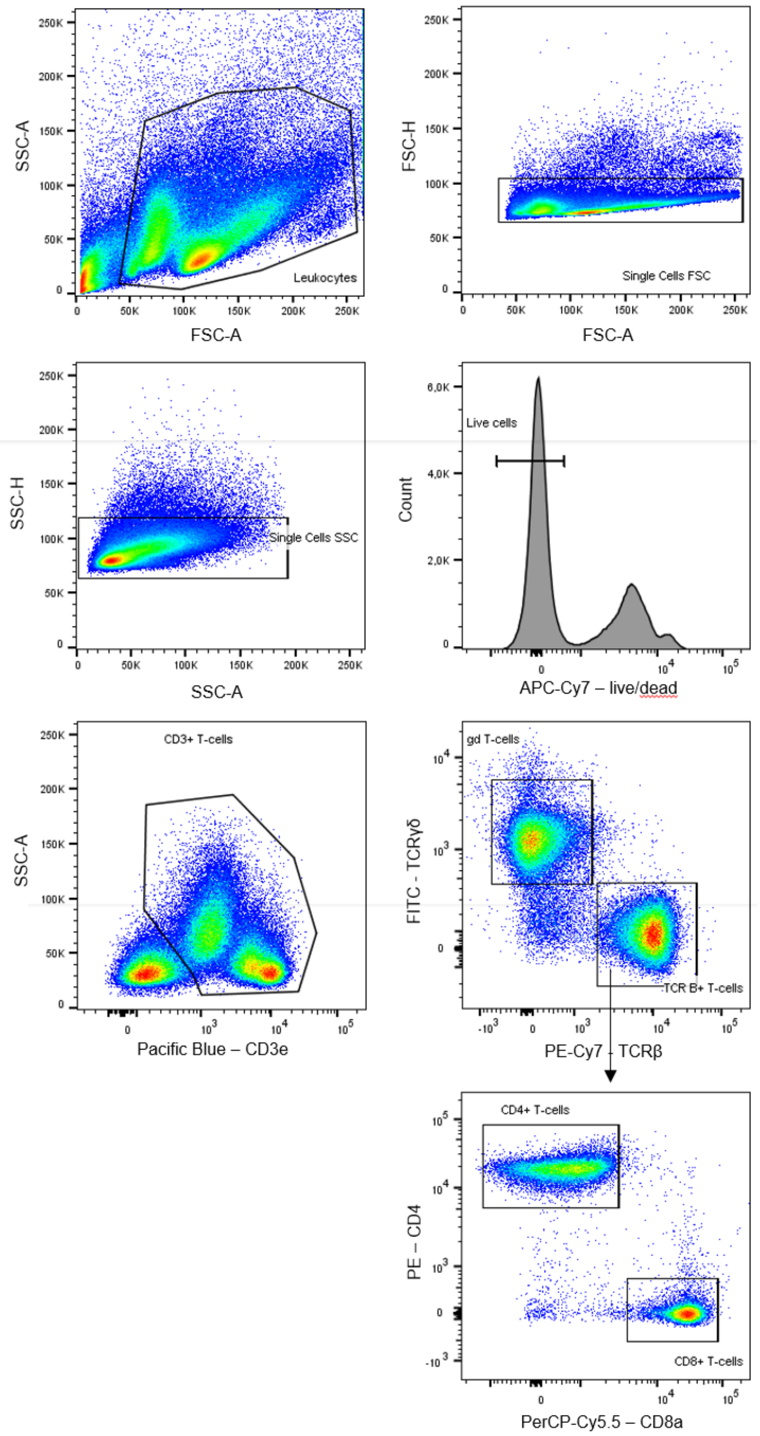

Supplementary Figure 10:

FACS gating strategy used for T-cell analyses of transplant experiments in Fig. 4C.

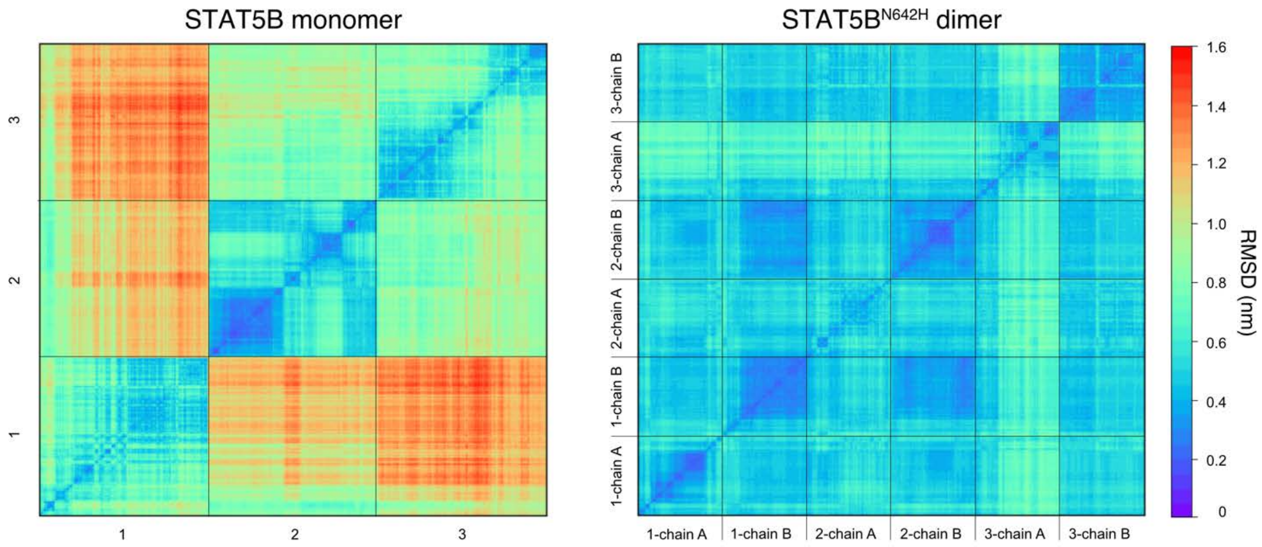

Supplementary Figure 11:

All-to-all root mean square deviation (RMSD) of the non-hydrogen atoms of the SH2 domain in the unphosphorylated STAT5B (left) and STAT5B^{N642H} antiparallel dimer (right). The RMSD is calculated between each conformation (every 2 ns) with respect to all other conformations visited in all the tested systems. The color scale is in RMSD (nm) and applies to both panels.

Supplementary Table 1. Data collection and refinement statistics (molecular replacement)

Dataset	STAT5B	STAT5B^{N642H}
**Space group**	P21212	P21212
**Unit cell dimensions**		
a (Å)	134.60	132.38
b (Å)	138.62	141.40
c (Å)	76.50	72.85
α, β, γ (°)	90,90,90	90,90,90
Wavelength (Å)	0.9792	0.9795
Resolution (Å)	69.31-3.29	70.7-3.21
Highest resolution bin (Å)	3.41-3.29	3.32-3.21
No. of reflections	139209	151137
No. of unique reflections	21967 (2135)	23029 (2257)
Completeness (%)	98.11 (98.48)	99.90 (100.0)
R_{merge}	0.09 (1.02)	0.11 (1.08)
I/σ (I)	12.9 (1.9)	13.8 (1.8)
Multiplicity	6.3 (6.9)	6.6 (6.7)
**Refinement**		
R_{work}	0.2664 (0.3445)	0.2653 (0.3889)
R_{free}	0.3057 (0.4000)	0.2863 (0.4170)
Average B-factor (Å2)		
Overall	109.20	86.67
From Wilson plot	114.30	95.95
**No. of atoms**		
Protein	7677	7897
Water	3	36
**RMS deviations from ideal**		
Bond lengths (Å)	0.004	0.006
Bond angles (o)	1.00	1.17
**Ramachandran outliers (%)**		
Residues in most favored regions (%)	92.46	91.75
Residues in allowed regions (%)	6.95	6.05
Outliers (%)	0.59	2.21

*Number of xtals for each structure = 1.

Supplementary Table 2. List of sustained inter-chain contacts in the unphosphorylated, antiparallel STAT5B^{N642H} dimer simulations. Data is presented as average contact frequency (%) over three independent systems ± standard error of the mean (SEM).

Chain A			Chain B		
**Residue**	**Domain**	**Contact frequency%±SEM**	**Residue**	**Domain**	**Contact frequency%±SEM**
GLU212	CCD	47± 27	GLU231	CCD	51 ± 26
GLN216	CCD	48 ± 28	GLN234	CCD	42 ± 23
GLU231	CCD	57 ± 29	LYS235	CCD	53 ± 28
GLN234	CCD	55 ± 29	GLN238	CCD	53 ± 27
PRO306	CCD	58 ± 29	PRO308	CCD	48 ± 24
GLU310	CCD	49 ± 24	GLU311	CCD	41 ± 20
GLU315	CCD	53 ± 27	MET312	CCD	42 ± 23
			GLU315	CCD	56 ± 29
			LYS384	DBD	45 ± 24
			PHE496	Linker	58 ± 30
			ALA497	Linker	59 ± 30
			VAL498*	Linker	87 ± 7
			PRO499	Linker	58 ± 30
			ASP500	Linker	58 ± 29
			LYS501*	Linker	83 ± 13
			GLY550	Linker	42 ± 21
			LEU551	Linker	48 ± 25
			SER552	Linker	53 ± 28

* present in all three systems.

Supplementary Table 3. Antibodies used for flow cytometry.

Antigen	Fluorochrome	Clone	Catalog No.
Fc-Block™	Unconjugated	93	14-0161-82 (eBioscience)
CD3e	eFluor® 450	eBio500A2	48-0033-82 (eBioscience)
CD90.2 (Thy1.2)	APC	53-2.1	17-0902-81 (eBioscience)
CD25	APC	PC61.5	17-0251-81 (eBioscience)
CD4	PE	GK1.5	12-0041-82 (eBioscience)
CD8a	PerCP-Cyanine5.5	53-6.7	45-0081-82 (eBioscience)
TCR β	PE-Cy™ 7	H57-597	560729 (BD Pharmingen)
TCR γδ	FITC	eBioGL3	11-5711-82 (eBioscience)
Ter119	APC- Cy™ 7	TER-119	116223 (Biolegend)
