# Supplementary material for: Structural and functional consequences of the STAT5BN642H driver mutation
Source: Nat Commun. 2019 Jun 7;10:2517. doi: 10.1038/s41467-019-10422-7 (PMC6555848; doi:10.1038/s41467-019-10422-7)
Supplement: Supplementary file 2 — Description of Additional Supplementary Files [file 41467_2019_10422_MOESM2_ESM.pdf]

Description of Additional Supplementary Files

File Name: Supplementary Movie 1

Description: The simulation trajectory for the STAT5B dimer (system 3) over 75 ns. The CCD (cyan), DBD (blue), linker domain (purple) and SH2 domain (orange) are all shown with residue N642 highlighted in green. A smoothing window of 5 frames was applied using VMD, and the protein was aligned to the conformation obtained after the equilibration simulations. Water and ions are not shown for clarity. As can be seen in the movie, the inter-monomer contacts are lost at ~75 ns, resulting in dissociation and dimer separation.

File Name: Supplementary Movie 2

Description: The final 75 ns of the 1 µs trajectory for the $STAT5B^{N642H}$ dimer (system 1). The color scheme is identical to Supplementary Video 1 with H642 highlighted in green. A smoothing window of 5 frames was applied using VMD, and the protein was aligned to the conformation obtained after the equilibration simulations. Water and ions are not shown for clarity. As can be seen in the movie, in contrast to the wild type STAT5B dimer, $STAT5B^{N642H}$ dimer remains stable for the entirety of the simulation.
