# Supplementary material for: Structural and functional consequences of the STAT5BN642H driver mutation
Source: Nat Commun. 2019 Jun 7;10:2517. doi: 10.1038/s41467-019-10422-7 (PMC6555848; doi:10.1038/s41467-019-10422-7)
Supplement: Supplementary file 5 — Reporting Summary [file 41467_2019_10422_MOESM5_ESM.pdf]

nature research

Corresponding author(s): Patrick Gunning, Richard Moriggl, Sirano Dhe-Paganon

Last updated by author(s): Apr 24, 2019

Reporting Summary

Nature Research wishes to improve the reproducibility of the work that we publish. This form provides structure for consistency and transparency in reporting. For further information on Nature Research policies, see Authors & Referees and the Editorial Policy Checklist.

Statistics

For all statistical analyses, confirm that the following items are present in the figure legend, table legend, main text, or Methods section.

n/a	Confirmed	
☐	☒	The exact sample size (*n*) for each experimental group/condition, given as a discrete number and unit of measurement
☐	☒	A statement on whether measurements were taken from distinct samples or whether the same sample was measured repeatedly
☐	☒	The statistical test(s) used AND whether they are one- or two-sided<br>*Only common tests should be described solely by name; describe more complex techniques in the Methods section.*
☒	☐	A description of all covariates tested
☐	☒	A description of any assumptions or corrections, such as tests of normality and adjustment for multiple comparisons
☐	☒	A full description of the statistical parameters including central tendency (e.g. means) or other basic estimates (e.g. regression coefficient) AND variation (e.g. standard deviation) or associated estimates of uncertainty (e.g. confidence intervals)
☒	☐	For null hypothesis testing, the test statistic (e.g. *F*, *t*, *r*) with confidence intervals, effect sizes, degrees of freedom and *P* value noted<br>*Give P values as exact values whenever suitable.*
☒	☐	For Bayesian analysis, information on the choice of priors and Markov chain Monte Carlo settings
☒	☐	For hierarchical and complex designs, identification of the appropriate level for tests and full reporting of outcomes
☒	☐	Estimates of effect sizes (e.g. Cohen's *d*, Pearson's *r*), indicating how they were calculated

Our web collection on statistics for biologists contains articles on many of the points above.

Software and code

Policy information about availability of computer code

Data collection	*Provide a description of all commercial, open source and custom code used to collect the data in this study, specifying the version used OR state that no software was used.*
Data analysis	*Provide a description of all commercial, open source and custom code used to analyse the data in this study, specifying the version used OR state that no software was used.*

For manuscripts utilizing custom algorithms or software that are central to the research but not yet described in published literature, software must be made available to editors/reviewers. We strongly encourage code deposition in a community repository (e.g. GitHub). See the Nature Research guidelines for submitting code & software for further information.

Data

Policy information about availability of data

All manuscripts must include a data availability statement. This statement should provide the following information, where applicable:

- Accession codes, unique identifiers, or web links for publicly available datasets
- A list of figures that have associated raw data
- A description of any restrictions on data availability

The atomic coordinates and structure factors (STAT5B PDB code: 6MBW, STAT5BN642H PDB code: 6MBZ) have been deposited in the Protein Data Bank, Research Collaboratory for Structural Bioinformatics, Rutgers University, New Brunswick, NJ (http://www.rcsb.org/). The source data underlying Figures 2b–d, 3a-h, 4b-d, 6a-b, 7d, 8a-c, and Supplementary Figures 1, 2, 4, 5, 6, 7 and 8a-b are provided as a Source Data file. Additional data for Figures 3a–d , 4d are available online [doi:10.5281/zenodo.2654892]. Other data are available from the corresponding authors upon reasonable request.

Field-specific reporting

Please select the one below that is the best fit for your research. If you are not sure, read the appropriate sections before making your selection.

☒ Life sciences ☐ Behavioural & social sciences ☐ Ecological, evolutionary & environmental sciences

For a reference copy of the document with all sections, see nature.com/documents/nr-reporting-summary-flat.pdf

Life sciences study design

All studies must disclose on these points even when the disclosure is negative.

Sample size	Experimental design and number of mice assessed were based on prior experience with similar models and provided sufficient statistical power to discern significant differences.
Data exclusions	No data were excluded from analyses.
Replication	All experiments were performed independently at least twice (as indicated in figure legends), with the exception of the in vivo transplant experiment which was only performed once, as a proof-of-principle experiment, for ethical reasons adhering to the 3R principle. All data obtained were found to be reproducible.
Randomization	Randomization was not applicable to this study, as no treatment groups were required and all samples/mice were treated equally in all experiments.
Blinding	Blinding was not possible for mice processing, due to disease severity.

Reporting for specific materials, systems and methods

We require information from authors about some types of materials, experimental systems and methods used in many studies. Here, indicate whether each material, system or method listed is relevant to your study. If you are not sure if a list item applies to your research, read the appropriate section before selecting a response.

Materials & experimental systems

n/a	Involved in the study	
☐	☒	Antibodies
☐	☒	Eukaryotic cell lines
☒	☐	Palaeontology
☐	☒	Animals and other organisms
☒	☐	Human research participants
☒	☐	Clinical data

Methods

n/a	Involved in the study	
☒	☐	ChIP-seq
☐	☒	Flow cytometry
☒	☐	MRI-based neuroimaging

Antibodies

Antibodies used	Polyclonal rabbit anti-phospho-STAT5 (Y694) (#71-6900; Invitrogen, Thermo Fisher Scientific; 1:1,000), monoclonal mouse anti-STAT5 (#610191; BD Biosciences; 1:1,000), monoclonal mouse anti-HSC70 (#sc-7298; Santa Cruz Biotechnology; 1:10,000), monoclonal mouse anti-α-tubulin (#sc-32293; Santa Cruz Biotechnology; 1:5000), monoclonal rabbit anti-CD3 (#RM-9107; Thermo Fisher Scientific; 1:300), monoclonal mouse anti-Ki67 (#NCL-Ki67p; Novocastra, Leica Biosystems; 1:1,000), mouse anti-CD16/CD32 (Fc-Block; Unconjugated, clone 93, Cat# 14-0161-82, eBioscience), mouse anti-CD3e (eFluor 450, clone eBio500A2, Cat# 48-0033-82, eBioscience), mouse anti-CD90.2 (Thy1.2) (APC, clone 53-2.1, Cat# 17-0902-81, eBioscience), mouse anti-CD4 (PE, clone GK1.5, Cat# 12-0041-82, eBioscience), mouse anti-CD8a (PerCP-Cyanine5.5, clone 53-6.7, Cat# 45-0081-82, eBioscience), mouse anti-TCR β (PE-Cy 7, clone H57-597, Cat# 560729, BD Pharmingen), mouse anti-TCR γδ (FITC, clone eBioGL3, Cat# 11-5711-82, eBioscience), mouse anti-Ter119 (APC-Cy 7, clone TER-119, Cat# 116223, Biolegend) and mouse anti-CD25 (APC, clone PC61.5, Cat# 17-0251-81, eBioscience).
Validation	Antibodies used in this study are routinely used by us and were previously published for their respective applications (Pham et al 2018, J Clin Invest, 128(1): 387-401). All FACS antibodies have validation information and publications listed on the manufacturer's websites.

Eukaryotic cell lines

Policy information about cell lines

Cell line source(s)	32D murine myeloid cells (#ACC 411) and Ba/F3 murine pro-B cells (#ACC 300) were purchased from the German Collection of Microorganisms and Cell Cultures (DSMZ).

October 2018

Authentication	Authentication was not performed on these cells as they are murine cells and were used within 6 months of purchase from the cell bank.
Mycoplasma contamination	32D and Ba/F3 cells tested negative for mycoplasma contamination during routine checks.
Commonly misidentified lines (See ICLAC register)	No commonly misidentified lines were used in this study.

Animals and other organisms

Policy information about studies involving animals; ARRIVE guidelines recommended for reporting animal research

Laboratory animals	Transgenic mice expressing either hSTAT5B or hSTAT5B N642H under the control of the Vav1 promoter were generated and bred on a C57BL/6N background (Charles River Laboratories), as previously described (Pham et al 2018, J Clin Invest, 128(1): 387-401). Mice were utilized between 7 and 10 weeks of age, as detailed in the figure legends. Both male and female mice were used in the analyses, as no gender bias was previously confirmed (Pham et al 2018, J Clin Invest, 128(1): 387-401). For transplant experiments, female C57BL/6N (Charles River Laboratories) mice were used as recipients.
Wild animals	The study did not involve wild animals.
Field-collected samples	The study did not involved samples collected from the field.
Ethics oversight	All animal studies were discussed and approved by the institutional ethics committee of the University of Veterinary Medicine Vienna, and animal experiment licenses were granted under GZ 68.205/0166-WF/V/3b/2015 and 68.205/0103-WF/V/3b/2015 (Austrian Federal Ministry of Science, Research and Economy). All animals were bred and maintained in a specific pathogen-free environment in the experimental mouse facility at the University of Veterinary Medicine (Vienna, Austria).

Note that full information on the approval of the study protocol must also be provided in the manuscript.

Flow Cytometry

Plots

Confirm that:

☒ The axis labels state the marker and fluorochrome used (e.g. CD4-FITC).

☒ The axis scales are clearly visible. Include numbers along axes only for bottom left plot of group (a 'group' is an analysis of identical markers).

☒ All plots are contour plots with outliers or pseudocolor plots.

☒ A numerical value for number of cells or percentage (with statistics) is provided.

Methodology

Sample preparation	For FACS of lymph nodes, single cell suspensions were made by mincing the tissue through a 70 µm cell strainer. Cells were stained for FACS analysis with primary antibodies for 30 minutes in the dark. Cells were washed 2x in cold PBS and samples were acquired. For organ infiltration analyses, whole body perfusion with phosphate-buffered saline (PBS) was performed on hSTAT5B N642H or WT mice, and organs were then collected and minced through a 70 µm cell strainer. Leukocytes were isolated using 40% and 78% Percoll gradients and were then stained for FACS analysis with primary antibodies for 30 minutes in the dark. Cells were washed 2x in cold PBS and samples were acquired. Counting beads were added before acquisition in order to quantify absolute cell numbers.
Instrument	All FACS analyses were performed on a FACSCanto II instrument (BD Biosciences).
Software	Acquisition of FACS data was performed using FACSDiva software (BD Biosciences). Further analyses were performed using FlowJo 10 software.
Cell population abundance	For transplant experiments, purity of the sorted gamma delta T-cell populations was determined by re-analysing sorted cells by FACS using identical gating strategies, and purity was found to be >90%.
Gating strategy	All FACS gates were kept identical for comparisons between all samples within a defined group of analyses (e.g. for each organ analysed). The starting cell populations were gated on the lymphocyte population from the FSC/SSC plot. These cells were then gated on single cells by doublet discrimination using both FSC-A/FSC-H and SSC-A/SSC-H. Single cells were gated on live cells based on negativity of a live/dead stain. Live cells were gated on CD3+ staining, and were then plotted against TCRgd and TCRB to gate on the respective single positive populations. Both the TCRgd+ and TCRB+ cells were then plotted against CD4 and CD8 to gate on and quantify single positive or double negative populations. Gates were determined and set based on plots obtained from unstained negative control samples.

☒ Tick this box to confirm that a figure exemplifying the gating strategy is provided in the Supplementary Information.
